# Genome Size, Cytotype Diversity and Reproductive Mode Variation of *Cotoneaster integerrimus* (Rosaceae) from the Balkans

**DOI:** 10.3390/plants10122798

**Published:** 2021-12-17

**Authors:** Faruk Bogunić, Sonja Siljak-Yakovlev, Irma Mahmutović-Dizdarević, Alma Hajrudinović-Bogunić, Mickaël Bourge, Spencer C. Brown, Edina Muratović

**Affiliations:** 1 Faculty of Forestry, University of Sarajevo, Zagrebačka 20, 71000 Sarajevo, Bosnia and Herzegovina; a.hajrudinovic@sfsa.unsa.ba; 2 Ecologie Systématique Evolution, Univ. Paris-Sud, CNRS, AgroParisTech, Université Paris-Saclay, CEDEX, 91405 Orsay, France; sonia.yakovlev@universite-paris-saclay.fr; 3 Laboratory for Research and Protection of Endemic Resources, Department of Biology, Faculty of Sciences, University of Sarajevo, Zmaja od Bosne 33-35, 71000 Sarajevo, Bosnia and Herzegovina; irma.m@pmf.unsa.ba (I.M.-D.); edina.muratovic@pmf.unsa.ba (E.M.); 4 Institute for Integrative Biology of the Cell (I2BC), CEA, CNRS, Univ. Paris-Sud, Université Paris-Saclay, CEDEX, 91198 Gif-sur-Yvette, France; mickael.bourge@i2bc.paris-saclay.fr (M.B.); spencer.brown@sfr.fr (S.C.B.)

**Keywords:** apomixis, Balkans, clonality, *Cotoneaster integerrimus*, cytotype, genome size, polyploidy

## Abstract

*Cotoneaster integerrimus* represents a multiploid and facultative apomictic system of widely distributed mountain populations. We used flow cytometry to determine genome size, ploidy level, and reproduction mode variation of the Balkan populations, supplemented by analysis of nuclear microsatellites in order to address: (i) geographic distribution and variation of cytotypes among the populations; (ii) variation of reproduction mode and the frequency of sexuality; (iii) pathways of endosperm formation among the sampled polyploids and their endosperm balance requirements; (iv) genotypic diversity and geographic distribution of clonal lineages of polyploids. The prevalence of apomictic tetraploid cytotype followed by sexual diploids and extremely rare triploids was demonstrated. This prevalence of tetraploids affected the populations’ structure composed from clonal genotypes with varying proportions. The co-occurrence of diploids and tetraploids generated higher cytotype, reproductive mode, and genotypic diversity, but mixed-ploidy sites were extremely rare. The endosperm imbalance facilitates the development and the occurrence of intermediate triploids in mixed-ploidy populations, but also different tetraploid lineages elsewhere with unbalanced endosperm. All these results showed that the South European populations of *C. integerrimus* have higher levels of cytotype and reproductive diversity compared to the Central European ones. Therefore, the South European populations can be considered as a potential reservoir of regional and global diversity for this species.

## 1. Introduction

Genome size, as a fundamental biological characteristic of evolutionary significance, has implications on overall biodiversity [1]. The causes of genome size variation in plants are varied, but the most striking is polyploidization. The evolutionary significance of polyploidy has tremendous and far-reaching implications for plant diversity [2]. It is assumed that almost 50% of plant groups have been affected by at least a single polyploidization event throughout their evolutionary history [3]. Polyploidy recomposes genome structure, alters gene expression, induces phenotypic and physiological changes, and provide adaptive potential to the polyploid plant [4,5,6]. Allopolyploidy is considered to be more frequent than autopolyploidy [7], but both processes profoundly shape plant diversity, either independently or in concert ([8], and references therein).

One of the consequences of being a polyploid is breakdown of self-incompatibility, allowing a shift towards asexual reproductive mode [9,10,11]. Apomixis (asexual seed formation) represents an effective life strategy by which apomicts preserve and maintain hybrid and heterozygous genetic lineages, cytotypes with unbalanced chromosomes, enabling their long-term persistence and dispersal [12]. Apomixis is highly correlated with polyploidy and hybridity. However, it is still unclear as to which of these factors precedes and what exact genetic and developmental mechanisms regulate the emergence of apomixis [13].

Gametophytic apomixis, the most common type of asexual reproduction, represents the formation and development of unreduced megagametophyte (parthenogenesis) coupled with the fertilization of unreduced central cell (pseudogamy) [14,15]. As a consequence of bypassing meiosis and lack of genome recombination, apomicts have a genetic structure identical to the maternal plant, thus creating clonal populations. Apomictic polyploids show a greater colonization ability by occupying more extreme ecological niches and persisting in larger distribution areas (geographic parthenogenesis, [14]) than their diploid sexual relatives [16,17,18]. Although genetically uniform, apomicts acquire genetic variation over time via accumulation of spontaneous mutations and via residual sexuality fostering diversity within clonal populations [19,20,21]. One of the mechanisms enhancing genetic diversity of apomictic populations comes from crosses between asexual and related sexual lineages, resulting in offspring that have a predominant apomictic mode of reproduction [17,22,23]. Given the long-life cycle of woody species, apomictic reproduction and independence of mates is extremely important in the early stages of the establishment of new populations and colonization of new environments.

Genus *Cotoneaster* Medik. (Rosaceae, Spiroaeoideae, Pyreae, [24]) includes shrubs and some small trees that are distributed in Europe, North Africa, and temperate areas of Asia (except Japan) [25]. The species number varies greatly among authors and ranges from 50–70 [26], to ≈90 [27], to ≈400 [25]. Evolutionary history and diversification of *Cotoneaster* have been affected by homoploid and heteroploid hybridization, as well as autopolyploidy and apomixis [25,28,29,30,31,32,33,34,35]. Both classical chromosome countings and cytometric analyses confirmed a series of ploidy levels in the genus ranging from diploids to hexaploids, with a prevalence of tetraploids.

Apomixis has been proven to be the most frequent reproductive mode in *Cotoneaster* that was primarily inferred from embryological data [36,37,38], breeding experiments [39], and later by molecular analyses [31,33]. Recently, apomixis has been confirmed using flow cytometry and different reproductive pathways of seed formation in tetraploids of *C. integerrimus* Medik. [29,30].

Key information on embryo sac development of several tri- and tetraploid *Cotoneaster* species (*C. rosea* Edgew., *C. nitens* Rehd. & Wils., *C. bullata* Bois, *C. obscura* Rehd. & Wils., *C. acutifolia* var. *villosula* Rehd. & Wils., *C. racemiflora* var. *soongorica* Schneid.) was provided in 1962 [36], and for *C. melanocarpus* Fisch. ex Blytt much later [37,38]. These species showed a strong tendency towards apomictic reproduction, namely, apospory and rarely diplospory, and the early development of their embryo sacs had general similarities with the other apomictic rosaceous genera. The most frequent development of unreduced embryo sac implied degeneration of the primary megaspore mother cell in the earliest developmental stages of the ovule and its replacement with unreduced embryo sac originating from nucellar cells (apospory). In several cases, the unreduced embryo sac developed from archesporial cells following the degeneration of the primary megaspore mother cell (diplospory). The secondary mother megaspore cell also occurred and was developed from unreduced embryo sac or via meiosis [36]. The reduced embryo sacs were rarely observed in the studied species [36,37,38]. The authors did not provide data on the polar nuclei. Conclusion of those findings is that most polyploid species of *Cotoneaster* are apomictic [25,32]. Sexual reproduction is less represented in *Cotoneaster* and follows a common pattern of development of *Polygonum* type of embryo sac: seeds of diploid mothers have diploid embryo and triploid endosperm; seeds of tetraploid mothers have tetraploid embryo and hexaploid endosperm [29,30].

In recent studies, only a tetraploid cytotype in *C. integerrimus* populations from Central Europe was found [30,40]. The same authors [30] proved that facultative apomixis was the main reproductive mode, followed by autonomous apomixis and haploid parthenogenesis, while the proportion of sexuality was 10% in the studied sample. The authors showed that different reproductive pathways involved the interaction of reduced and unreduced gametes documented in both sexuals and asexuals.

In this study, we analyzed the variation of genome size, ploidy level, and the diversity of reproductive modes of the *C. integerrimus* populations from the Balkans with additional samples from several other European regions. In addition, molecular analyses based on nuclear microsatellite markers were performed on the studied cytotypes. Our questions aimed to assess: (i) the geographic distribution and variation of cytotypes within populations in the sampled area; (ii) the most prevalent mode of reproduction in polyploid cytotypes and the frequency of sexuality; (iii) the pathways of endosperm formation among the sampled polyploids and their endosperm balance requirements; (iv) genotypic diversity and geographic distribution of clonal lineages of polyploids. Finally, we discuss the importance of South European populations of *C. integerrimus* within the context of cytotype diversity and variation in reproduction mode.

## 2. Results

### 2.1. Genome Size and Ploidy Level Variation

**Figure 1:**
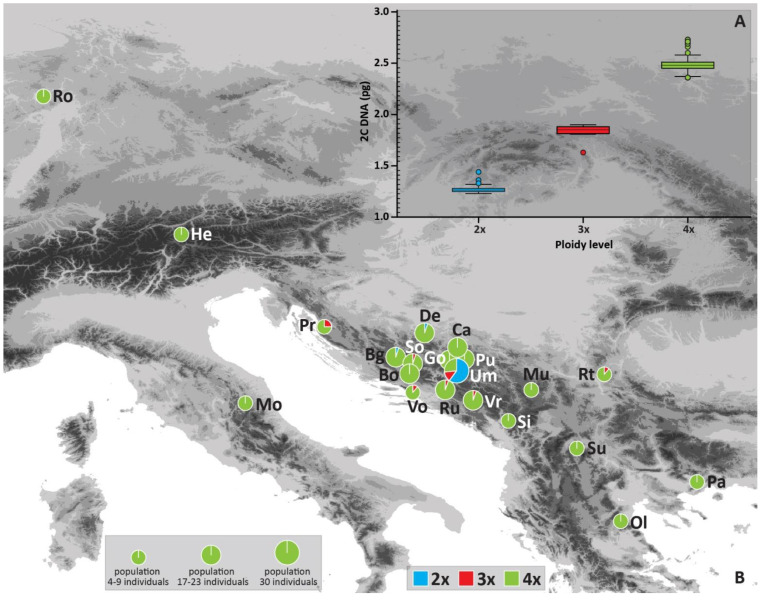
Absolute nuclear genome size (2C) of diploid, triploid, and tetraploid cytotypes (**A**) (boxes define 95th percentile interval; horizontal lines indicate mean value; whiskers indicate standard deviation: outliers are shown as circles), and geographical distribution of *Cotoneaster integerrimus* cytotypes and their frequency (%) per site/population (**B**). Site abbreviations (Site IDs) correspond to Table 2.

The flow cytometry (FCM) of 208 *Cotoneaster integerrimus* individuals from Bosnia and Herzegovina resulted in three distinct groups whose holoploid genome sizes (2C) corresponded to three different ploidy levels, namely, di-, tri- and tetraploid cytotypes (Table 1, Figure 1A). The two singular values corresponded to pentaploid (2C = 2.99 pg, 1Cx = 0.598 pg) and hexaploid cytotype (2C = 3.35 pg, 1Cx = 0.558 pg); thus, both values were excluded from further analyses. The 2C genome size mean values were 1.28 pg in diploids, 1.856 pg in triploids, and 2.481 pg in tetraploids. The significantly (F_2, 207_ = 10.98, *p* > 0.001) highest monoploid (1Cx) genome size value was recorded in diploid, and the lowest in triploid cytotype (Table 1).

Geographic distribution of cytotypes showed a clear prevalence of tetraploids (89% in the total sample) in each population except the Umoljani (Um) in Bosnia and Herzegovina (Table 2, Figure 1B). The sample for Figure 1B encompassed the ploidies obtained from flow cytometry as well as the estimated ploidy levels from microsatellite data for 65 individuals (explained in Table 2 and the Material and Methods section). Diploids (7.3% in total sample) were recorded in only three populations with a single individual in Borova glava (Bg) and Devečani (De), and 18 individuals in Umoljani (Um) (Table 2, Figure 1B). Triploids (3.7% in total sample) were observed in seven populations and were represented with just one individual in six populations (Vrbe-Vr, Rujište-Ru, Sovićka vrata-So, Vošac-Vo, Premužićeva staza-Pr, Rtanj-Rt) and four individuals in the population Umoljani (Table 2).

### 2.2. Flow Cytometric Seed Screening

Only unambiguous results of flow cytometric seed screening (FCSS) are presented. Approximately one quarter of analyzed seeds had minor additional peaks corresponding to doubled nuclear DNA levels mirroring endoreplicated embryo and/or endosperm nuclei. In total, the reproductive mode was characterized for 591 seeds from three *C. integerrimus* cytotypes (Table 2).

**Figure 2:**
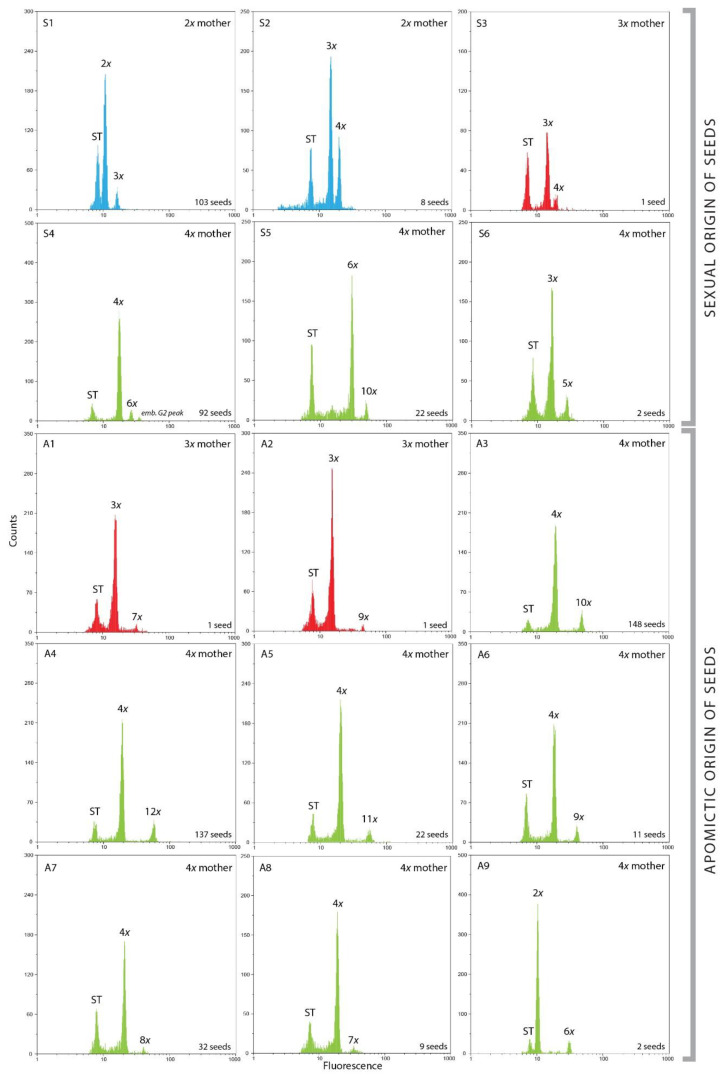
Flow cytometric histograms (log abscissa) of *Cotoneaster integerrimus* seed formation from sexual (**S1**–**S6**) or asexual (**A1**–**A9**) origin. The first fluorescence peaks correspond to the internal standard (ST, *Oryza sativa* L. ssp. *japonica* ‘Nipponbare’), the second ones to the embryo, and the third ones to the endosperm (blue: 2x mother, red: 3x mother, green: 4x mother).

In general, inferred pathways of sexual and asexual seed formation showed great diversity and included both reduced and unreduced gametes within each cytotype (Figure 2, Table 3). The tetraploid cytotype was the most abundant, and it consequently resulted in the highest number of reproductive pathways, predominantly apomictic. Diploids involved only sexual reproduction (Table 3). The triploid cytotype was represented with only three seeds, one of sexual and two of apomictic origin (Table 3). In any case, sexual and asexual seed formation was clearly distinguished.

#### 2.2.1. Seed Origin in Diploids

Diploids yielded exclusively sexually originated seeds having the same or increased ploidy level compared to the mother plant, which depended on sperm cell ploidies (Figure 2(S1,S2), Table 3). The profile 2x emb.:3x end. was as expected the most represented (103 seeds) and included both reduced female and male gametes. The profile 3x emb.:4x end. (eight seeds) was a result of interploid crosses between diploids and tetraploids. Those seeds originated from joint of reduced gametes (2x) from tetraploid males and a reduced egg cell (1x) of diploid mother.

#### 2.2.2. Seed Origin in Triploids

All three analyzed triploid seeds had different profiles (Figure 2(S3,A1,A2), Table 3). The only sexual profile 3x emb.:4x end. from one seed included 2x male gamete and reduced (1x) egg cell (Table 3). The other two seeds were of apomictic origin. One included unreduced egg cell fecundated with reduced (1x) sperm cell creating 3x emb.:7x end. Profile, and the other included unreduced (3x) sperm cell creating 3x emb.:9x end. profile (Figure 2(A1,A2), Table 3).

#### 2.2.3. Seed Origin in Tetraploids

Three pathways of sexual seed formation were observed in tetraploids (Figure 2(S4–S6), Table 3). The most frequent profile 4x emb.:6x end. (92 seeds) was formed by fertilization of a meiotically reduced egg cell (2x) with reduced 2x sperm cell (Figure 2(S4)). Fecundation of unreduced female gamete (4x) with reduced 2x male gamete resulted in the formation of B_iii_ hybrids (6x emb.:10x end.) and was detected in 22 seeds (Figure 2(S5)). Two cases of gamete exchange between cytotypes were registered. The fecundation of a reduced egg cell (2x) of tetraploid female with reduced gamete (1x) of diploid male resulted in a 3x emb.:5x end. profile (Figure 2(S6), Table 3).

Pseudogamous seed formation had various pathways. The two most frequent apomictic profiles were 4x emb.:10x end. (148 seeds, Figure 2(A3)) and 4x emb.:12x end. (137 seeds, Figure 2(A4)). In the first case, unreduced polar nuclei were fertilized with reduced 2x male gametes (or, less likely, two 1x gametes). The second case included fecundation of unreduced polar nuclei with two 2x male gametes (Table 3). The following profiles were observed with lower frequencies (Table 3): 4x emb.:11x end. (22 seeds), 4x emb.:9x end. (11 seeds), 4x emb.:7x end. (9 seeds). The 4x emb.:11x end. profile included the fecundation of polar nuclei with 3x pollen or simultaneously with 2x and 1x sperm cells (Figure 2(A5), Table 3). The 4x emb.:9x end. (Figure 2(A6)) profile resulted from the fecundation of polar nuclei with 1x sperm cell. Exact pathway of origin for the 4x emb.:7x end. profile (Figure 2(A8)) remained unresolved, as well as several profiles with oddly high ploidies of endosperm (14x, 15x, 16x, 20x, data not shown).

Moreover, profiles that corresponded to autonomous apomixis (4x emb.:8x end.) have been documented in 32 seeds (Figure 2(A7)). Finally, two seeds originated via haploid parthenogenesis (2x emb.:6x end.) were observed. These cases involved reduced (2x) sperm cells fecundating solely central nuclei (Figure 2(A9), Table 3).

#### 2.2.4. Endosperm Balance

All sexually derived seeds that involved the exchange of reduced gametes within diploid and tetraploid cytotypes had a balanced endosperm with a maternal to paternal genome ratio of 2m:1p (Table 3). Unbalanced endosperms with a ratio of 1m:1p and 4m:1p were observed in sexually produced seeds originating from interploid crosses between diploids and tetraploids (Table 3). On the other hand, within the sample of apomictically originated seeds (354 seeds), the apomictic profile 4x emb.:12x end. was the only one with the balanced endosperm (137 seeds, 38.7%). The remaining seeds of apomictic origin (217 seeds, 61.3%) had unbalanced endosperms (Table 3). Interestingly, the same tetraploid mother plants produced sexual and apomictic seeds having both balanced and unbalanced endosperm (Table S1).

### 2.3. Genotypic Variation in Cotoneaster integerrimus Populations

Five microsatellite loci were successfully amplified in the analyzed *C. integerrimus* populations and yielded a total of 99 alleles from 254 individuals (Table S2).

A total of 92 multilocus genotypes (MLGs) were obtained from five loci (Ng, Table 4). Each of the studied diploids (N = 13) had a unique genotype (Table S2). The clonal genotypes (N = 32) were found within a polyploid pool of 241 individuals (Table S2). Each population contained at least one clonal genotype shared by a different number of individuals within a population, indicating clonal reproduction (Ni = 194, Table 4). The proportion of the detected clones (Ng/N) ranged from 0.1 to 0.91 (Table 4), reflecting the different level of clonal structure of populations. In each population, at least two individuals shared the same genotype (Table 4). Moreover, certain populations were completely clonal (Pu–Mt. Trebević, Si-Sinjajevina, He–Austria; Ng = 1, Table 4). In the majority of populations, the effective number of genotypes was higher than one, indicating the presence of unique genotypes in populations (Table 4). Different values of genotypic diversity of populations (Table 4) showed their diverse structure of clonal and unique genotypes, suggesting different ratios of sexuality and asexuality in populations.

While the majority of clonal genotypes (N = 29) were geographically restricted within a single or two populations, the two clonal genotypes occurred at multiple sites (Table S2). The more widespread clonal genotype occurred in populations Bo–Borova glava (N = 20), Go–Mt. Igman (N = 9), Ru–Rujište (N = 9), So–Sovićka vrata (N = 2), and Um–Umoljani (N = 1), which was equivalent to 17% of all polyploids. The second clonal genotype was less widespread and was shared by plants from three populations: So–Sovićka vrata (N = 4), Um–Umoljani (N = 1), and Vr–Vrbe (N = 3). One hundred and thirty-seven (56.8%) individuals belonged to 25 spatially limited clonal genotypes, while other individuals possessed unique genotypes.

**Figure 3:**
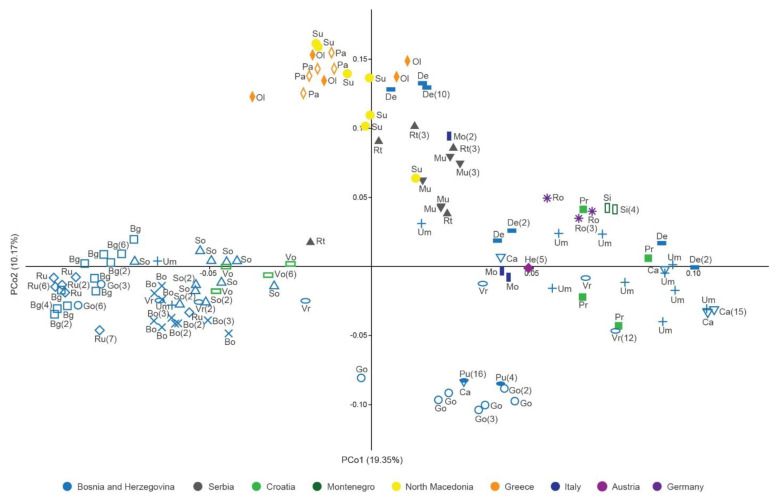
Principal coordinate analysis of the Jaccard distance matrix of multilocus genotypes of *Cotoneaster integerrimus* polyploids according to nuclear microsatellite data. Population abbreviations correspond to those in the Table 4. The numbers in the brackets denote the number of clones.

Principal coordinate analysis (PCoA) based on Jaccard distances was applied on *C. integerrimus* polyploid MLGs (Figure 3). PCoA revealed the pattern of grouping of MLGs dispersed along the first (PCo1) and the second (PCo2) coordinates (Figure 3). A weak regional geographic pattern of separation among MLGs groups was evident. The MLGs from western Balkans formed three groups along the PCo1: the first group consisted of MLGs from Bosnia and Herzegovina (Bg, Bo, Ru, Go, So, Um, Vr) and Croatia (Vo); the second consisted of several Bosnian MLGs (Go and Pu); and the third one was the most heterogeneous and consisted of MLGs originating from Bosnia and Herzegovina (Ca, De, Pr, Um, Vr), Croatia (Pr), and Montenegro (Si). MLGs from Italy (Mo), Austria (He), and Germany (Ro) were intermingled with Balkan MLGs. The southern Balkan MLGs from Greece (Ol and Pa) and North Macedonia (Su) clustered into a separate group along the second coordinate (Figure 3). Serbian MLGs (Mu and Rt) formed a group neighboring the southern Balkan cluster. Certain MLGs from De and Um populations were interspersed within the southern Balkan cluster.

## 3. Discussion

### 3.1. Genome Size Variation and Geographic Distribution of Cotoneaster integerrimus Cytotypes

*Cotoneaster integerrimus* showed stability of the monoploid genome size of three cytotypes, despite asymmetric sample size and significant differences between them. Monoploid values were quite similar to those obtained by Macková et al. [30] and Kšiňan et al. [40]. A slight decrease of monoploid genome size of polyploids compared to diploids was evident. Genome downsizing is a common phenomenon during polyploidization and inter-cytotype hybridization [42,43,44]. The obtained genome sizes and ploidies of seed embryos were consistent with leaf data. However, certain inconsistencies were present in precise determination of endosperm ploidy, which was calculated using the monoploid genome size of embryo. This issue has been often reported in studies dealing with flow cytometric seed screen [22,30]. The source of endosperm variation lies in possibility of the central cell’s fertilization by unbalanced and aneuploid gametes of variable genome size and in different number of nuclei in the central cells [22,23,45,46]. However, the observed variation in endosperm ploidy did not hinder the discrimination between the sexual and asexual origin of seeds but, in some cases, rather made it difficult to accurately infer the number and ploidy of sperm cells involved in the fertilization of central cell’s nuclei.

*Cotoneaster integerrimus* tetraploids are the dominant cytotype in the studied Balkan populations (Figure 1B), as also documented in Central Europe [30,40]. Populations from Central Europe were homogeneously tetraploid. Sexual diploids are detected only in the Western Alps [30]. In comparison, our study showed the greater presence of sexual diploids in the Balkans but stressed their rare occurrence as well. They were registered at the three populations in Bosnia and Herzegovina, of which two contained only one diploid individual. In contrast, it was the prevailing cytotype at the most numerous Umoljani population, outnumbering the polyploids (Figure 1B, Table 2). The observed co-occurrence of tetraploids and triploids in seven populations (Figure 1B) implicate the exchange of reduced gametes between tetraploid and diploid cytotypes. However, the sympatry of diploids, triploids, and tetraploids was documented only in the population Umoljani. We assume that diploids in other populations simply were not covered by sampling due to their overall rarity. Hence, a more extensive sampling strategy should be applied to test this assumption. If we consider the present results jointly with Macková et al. [30] and Kšiňan et al. [40], as a comprehensive dataset on *C. integerrimus* ploidy variation, it is apparent that diploids are a minority cytotype in the overall cytotype structure of the species. Diploids of *C. integerrimus* as a rare cytotype never form monoploid populations [47]. The coexistence of diploid and tetraploid cytotypes is also extremely rare in *C. integerrimus*. Diploid cytotype at the Umoljani site contributes markedly to ploidy richness, reproductive mode variation, and genotypic diversity. Namely, diploids affect cytotype dynamics through reproductive interactions with sexual and asexual tetraploids, generating intermediate triploid cytotype (Table 3). Furthermore, interploid crossings contribute to greater diversity of reproductive pathways, which was confirmed at the Umoljani site (Table S1). Finally, gene flow between di- and tetraploid cytotypes contributes to the higher genotypic diversity (Table 4).

The question remains as to why *C. integerrimus* diploids are so rare. Geographic distribution of *C. integerrimus* diploids in our sample confirmed a restricted and smaller geographic range relative to asexual tetraploids, which is a common pattern in different agamic groups [47,48,49,50]. These areas inhabited with diploids are considered as relict habitats in glacial refugia during the Pleistocene climate changes [10,51]. This assumes that the site Umoljani served as refugium for the persistence of diploids during glacial cycles. Such a scenario seems plausible for Umoljani as it is situated just above the huge Rakitnica gorge within the high mountains in Bosnia and Herzegovina, which has been confirmed as a refugium for many plant species [52]. Moreover, the Umoljani site has been identified as one of the hotspots of *Sorbus* cytotype diversity in the Balkans [23]. The current coexistence of the *C. integerrimus* diploids and tetraploids reflects the primary contact zone of cytotypes that involved in situ ancient formation of tetraploids via autopolyploidy or postglacial cytotype overlap of the two cytotypes (secondary contact zone). Nuclear microsatellites showed genetic divergence between diploids and tetraploids (Figure S1) and rather support the secondary contact zone. However, due to the small sample size and occurrence of clonal genotypes (six out of eight individuals) at the Umoljani site, it remains uncertain as to whether the current coexistence resulted from the primary or secondary contact. Such sites, where different sexual and asexual cytotypes coexist and interact, encouraging the formation of novel biodiversity, represent areas of particular conservation concern [53].

### 3.2. Asexual Seed Formation in Monoploid and Mixed-Ploidy Populations

Flow cytometric seed screening unequivocally confirmed pseudogamous apomixis as the most prevalent mode of reproduction in the *C. integerrimus* complex along the sampled area. While diploids are exclusively sexual, tetraploids combine asexual and sexual reproduction. Our results for tetraploids are fully consistent with those of Macková et al. [30], as well as with other related genera: *Amelanchier* [54], *Crataegus* [55], *Potentilla* [45], *Rubus* [56], and *Sorbus* [22,23]. All *C. integerrimus* tetraploids in monoploid populations showed similar patterns of seed formation involving both reduced and unreduced gametes (Table 3). Seed formation in tetraploids mainly involved central cell fertilization with one or two 2x sperm cells (two dominant profiles: 4x emb.:10x end. and 4x emb.:12x end., Table 3). The latter profile suggested dispermy of the central cell during endosperm formation [54,55,56], rather than fertilization with unreduced sperm cells. Namely, we did not notice the occurrence of male unreduced gametes in any of the supposed reproductive pathway of tetraploids (Table 3). The 10x endosperm was prevalent in *Crataegus* and *Rubus* but quite rare in *Amelanchier* and *Sorbus*, having 12x endosperm. Both 10x and 12x endosperm had almost equal frequency in *C. integerrimus* tetraploids, as confirmed by Macková et al. [30]. In addition, both ploidy levels of endosperm were present in seeds produced by the same plants, even yielding sexually originated seeds with balanced endosperm at the same time (Table S1). The capability of a single plant to simultaneously produce pseudogamous seeds with balanced/unbalanced endosperm, as well as seeds of sexual origin with a balanced endosperm, represents an advanced reproductive strategy. The potential to use the full benefits from both reproduction modes, accompanied with self-compatibility and self-pollination (10, 12), makes the *Cotoneaster integerrimus* tetraploid apomicts ecologically superior for rapid adaptation, colonization, and long-term persistence of populations in dynamic environments. The prevalence of tetraploid apomictic populations over diploid sexual ones goes in favor of geographic parthenogenesis along the sampled area (14).

The frequency of endosperm with odd ploidy levels was low (8.8%) in tetraploids of *C. integerrimus*, although this is common in related genera [22,23,30,53,55,56]. Such endosperm in our case would require the participation of triploid and haploid pollen that was not observed in the field, except for the population Umoljani. Lepší et al. [22] showed that the exact embryo and endosperm genome sizes primarily depend on the combination of actual gamete genome sizes involved in the fertilization and may markedly deviate from expected mean values, making profile interpretation difficult. We assume that these profiles with an unbalanced endosperm (4x emb.:7x end., 4x emb.:9x end. and 4x emb.:11x end.) represent pseudogamy that most likely included aneuploid sperm cells with variable genome size [55,56] or products of an irregular pseudogamous process [45]. However, our data showed that tetraploid cytotype apparently tolerate endosperm imbalance, but the relationship between endosperm imbalance tolerance and seed viability and germination remains uninvestigated.

In addition to pseudogamous development, we also observed a profile corresponding to autonomous apomixis (4x emb.:8x end.) (Table 3). Unlike Macková et al. [30], in our dataset, we detected some cases of G2 peaks of tetraploid embryos (Figure 2(S4)), and therefore we cannot precisely distinguish endosperm with endoreduplicated embryos. Finally, several cases of haploid parthenogenesis have been found in tetraploid seeds as a distinct rare pathway of asexual seed formation yielding unviable seeds and anormal development of the plant.

### 3.3. Sexuality in Cotoneaster integerrimus Populations

The fertilization via reduced (2x) gametes, resulting in seeds with a balanced endosperm (2m:1p), represents the main (19.4%, N = 92, Table 3) sexual reproductive pathway of tetraploid *C. integerrimus*, which is noticeably higher than in Central European populations (6.3%, [30]). As mentioned earlier, different seed formation pathways (apomictic and sexual) involving different combinations of gamete number can operate in one plant. On the other hand, the rate of sexuality within a single plant/population greatly varies, as evidenced by the proportion of clonal genotypes and complete clonality in some populations (Table 4).

The frequency (N = 22, 4.2%, Table 3) of unreduced female gametes (4x) in crosses with reduced male gametes (2x) resulted in B_iii_ hybrid seeds, with a similar proportion found in Central European populations. Our screening revealed only one fruitless hexaploid individual in the field, and we assume that this cytotype is extremely rare. The viability of B_iii_ hybrid seeds and seedlings is most likely low due to endosperm imbalance [22].

Sexuality in *C. integerrimus* also included bidirectional intercytotype crosses, resulting in 12 seeds with triploid embryos that accounted for ≈9% of all analyzed seeds from the Umoljani population. We expected that the number of such crosses would be much higher in a larger sample. Singular occurrence of triploid individuals in other populations (So, Ru, and Vr) also requires more extensive sampling to depict cytotype structure in populations. Our results provide evidence that gene flow occurs between diploid and tetraploid cytotypes in their sympatry, but the small number of triploids that have a weak or no fruit yield suggest a lower fitness than either parent (only three fertile pyrenes from two individuals). The occurrence of triploid cytotype in mixed-ploidy populations indicates the existing weak reproductive barriers between diploid and tetraploid cytotype, which nevertheless allow the formation of triploids but with a low frequency.

The key reproductive isolation mechanism in the formation of triploids is considered to be the triploid block, which operates in different polyploid systems [22,57,58,59,60]. The triploid block is caused by malfunction of the endosperm as a result of an imbalance between paternally and maternally imprinted genes during the endosperm development, and often leads to seed abortion [61]. The observed endosperm imbalance in all triploid seeds originated from di-, tri-, and tetraploid mother plants in the present dataset (Table 3), suggesting that a small portion of seeds are insensitive to the triploid block. These seeds, originated via intercytotype crosses in *C. integerrimus*, represent novel polyploid forms of genomic variation with potential for divergence. The frequency of repeated crosses between diploids and tetraploids accompanied by the relaxed endosperm balance would directly affect the frequency of triploids in mixed-ploidy populations. Although triploids are considered unstable and less fertile relative to diploids and tetraploids in Rosaceae [55,62], they play an important role as intermediates in the formation of tetraploids via ‘triploid bridge’ [23,59,63]. Our scenario involves bidirectional crosses between a sexual diploid cytotype and a predominantly apomictic tetraploid cytotype that produces a mostly apomictic triploid progeny of *C. integerrimus*. The reproductive success and persistence of the triploid cytotypes will primarily depend on the capabilities provided by their mating system [10]. Certainly, a future sampling design will encompass a larger geographic distribution of *C. integerrimus* populations and a larger number of individual plants/progenies to answer the question on the occurrence, dynamics, and distribution of triploids and their potential role in evolution of this complex.

### 3.4. Patterns of Genotypic Diversity in Cotoneaster integerrimus

The use of nuclear microsatellite markers confirmed cytometric results and shed a new light on the breeding system of *C. integerrimus*. The patterns of genotypic diversity within and among populations primarily reflected on the breeding system of *C. integerrimus* that favors pseudogamous reproduction. Favored asexuality is evident in the low level of genotypic diversity and the high proportion of clonal genotypes in most populations. While the studied diploids have unique MLGs, a trait of exclusive out-crossers [63,64], polyploids share the same genotypes in each population. Heteroploid populations showed higher levels of genotypic diversity. These patterns may reflect ancient hybridization between cytotypes that has taken place over time or current processes in which younger apomictic lineages tend to prevail via intercytotypic crosses [18,65]. The proportion of sexuality of tetraploids significantly differs among the populations, but the factor that governs the sexual reproduction in *C. integerrimus* remains unclear. On the other hand, the sexual reproduction of facultative apomicts may depend on changes in environmental conditions [56]. The interaction between the breeding system and environmental conditions allows for the facultative apomict to increase the rate of sexuality at a given time, reshuffling the overall genetic variability of populations in dynamic environments [66]. In any case, sexuality is an important and propulsive agent of genetic variability in monoploid and mixed-ploidy *C. integerrimus* populations. Additional factors contributing to genetic variability in different apomictic systems are mutations and residual recombination of the female gametophyte [20,63,67,68], but these aspects are beyond the scope of this study.

Although the most clonal MLGs were spatially restricted within the populations, few were dispersed among the populations. Moreover, the most widespread clonal MLG constituted the complete structure of the Bg population, and significantly in the Go and Ru sites. Geographical distances between the sites/populations with the clonal MLGs ranged between 40 and 70 km. The wide geographic occurrence of a certain clonal lineage confirmed its high abilities to colonize and spread in new geographic areas. This pattern might fit the idea of generalist clonal genotypes that are geographically widespread and occur in different habitats [18]. The widely distributed clonal MLGs may represent old *C. integerrimus* lineages that had the chance for multiple long-distance dispersal events, and spatially restricted ones may represent relatively new clonal genotypes that have not had time to disperse across environments [65]. In that context, the lack of clear geographic structure among the polyploid MLGs, as revealed by PCoA, might lie in their multiple origins via recurrent mating between widely distributed lineages and local genotypes, as well as multiple colonization of sites by different clones [18,68]. In such a scenario, recurrent admixture of divergent genotypes and their interaction will blur the geographic structuring of populations.

## 4. Materials and Methods

### 4.1. Plant Material

We sampled a total of 18 natural populations of *C. integerrimus* originating from the Balkans (covering Bosnia and Herzegovina and the neighboring regions of Serbia, Montenegro, Croatia, North Macedonia, and Greece (Figure 1A, Table 2)). Our sample was complemented with three populations from Italy, Austria, and Germany. More detailed sampling was performed in 10 Bosnia and Herzegovina populations encompassing 17–30 individuals per site, depending on the population size (Table 2). The distance between adult plants at all sites was at least 20 m to avoid collecting ramets. Altogether, 254 adult individuals from 21 populations were used for genome size, ploidy level determination, and molecular analysis. To determine the reproductive mode variation, we studied 591 seeds from the 63 mother individuals. The vouchers were deposited in the Herbarium of the National Museum of Bosnia and Herzegovina (SARA herbarium, voucher numbers 51809-51818).

### 4.2. Genome Size and Ploidy Level Determination

Absolute genome size and ploidy level determination was performed by flow cytometry (FCM) for 208 individuals from Bosnia and Herzegovina. Genome size determination followed the protocol previously used for *Sorbus* spp. [23]. Briefly, parts (around 1 cm^2^) of *C. integerrimus* fresh leaves and leaf material of internal standard (*Solanum lycopersicum* cv. Montfavet 63-5, 2C = 1.99 pg, [69]) were chopped together using a razor blade in cold Gif Nuclear Buffer [70]. The suspension was filtered through a 50 µm nylon mesh (CellTrics from Partec-Sysmex, Goertliz, Germany), and RNAse (Roche CustomBiotech, Mannheim, Germany) was added to 2.5 U mL^−1^. The nuclei were stained with propidium iodide (Sigma-Aldrich, Saint-Louis, United States of America) to a final concentration 50 µg mL^−1^ and incubated on ice for at least five minutes. The fluorescence of 5000 nuclei was recorded for each sample using a Partec CyFlow SL3 (Partec, Münster, Germany) 532 nm laser cytometer. Fluorescence histograms were analyzed using FloMax ver. 2.8 (Partec, Münster, Germany). The absolute 2C DNA values of *Cotoneaster* individuals were obtained by calculation of linear relationship between the fluorescence signals of unknown sample and known internal standard. Individual DNA ploidy levels [71] were inferred following earlier chromosome counts on *Cotoneaster* species and compared with obtained 2C DNA values. Each individual was assigned into particular cytotype: di-, tri-, and tetraploid. The total 2C DNA value/ploidy level ratio was used to obtain monoploid genome size (1Cx, [72]) of each cytotypes. The mean values of monoploid genome size of cytotype groups were tested using one-way ANOVA followed by Tukey’s HSD test. Prior to ANOVA, homogeneity of groups’ variances using Levene’s test and data distribution using Kolmogorov–Smirnov test were checked. Analyses were conducted using PAST 3.17 [73].

For individuals sampled outside Bosnia and Herzegovina (N = 65), the ploidy level was inferred from microsatellite data. The maximum number of alleles per locus was used to determine the ploidy level for those samples. This procedure was successfully applicable in certain groups [72,74,75,76], but this criterion was reliable for tetraploids only.

### 4.3. Flow Cytometric Seed Screening

Flow cytometric seed screening was successfully conducted on 591 seeds following the procedure described by Hajrudinović et al. [23]. Seeds (pyrenes) were collected from attached fruits on 14 diploid, 3 triploid, and 63 tetraploid mother individuals, previously cytotyped (Table S1). Only well-formed seeds, dried for 48 h at room temperature and kept in paper bags at 4 °C prior to analysis, were used. Each seed was analyzed separately. Only three healthy seeds were recovered from triploid plants, which indicates a high level of sterility of this cytotype. Entire seeds were co-chopped with part of fresh leaf (around 0.5 cm^2^) of internal standard *Oryza sativa* ssp. *japonica* cv. Nipponbare (2C = 0.9 pg, [77]) in cold Gif Nuclear Buffer following the steps in procedure described for genome size and ploidy level determination. Both linear and log histograms were recorded after the fluorescence was divided between two photomultipliers with a 50/50 mirror. Endosperm ploidy was calculated using the inferred monoploid genome size of embryo of the same seed. Estimated DNA ploidies of embryo and endosperm were compared to distinguish between sexual and apomictic origin of each analyzed seed and to deduce fertilization pathways according to [23,55,78].

### 4.4. Analysis of Nuclear Microsatellites

A modified CTAB-procedure [79] was used to extract total genomic DNA from around 20 mg of silica-dried leaf material. Amplification of five nuclear microsatellite markers was performed for 254 individuals (Table 2) following Robertson et al. [64]. The applied primers are primarily designed for *Malus × domestica* (CH01H10, CHO1FO2, and CHO2D11, [80]) and *S. torminalis* (MSS5 and MSS16, [81]) but were successfully applied in different rosaceous genera. An ABI PRISM 3500 Genetic Analyzer (Applied Biosystems, Foster City, CA, USA) was used for electrophoretic separation of PCR products. Alleles were sized relative to the internal size standard TAMRA 500 (Applied Biosystems, Warrington, United Kingdom). Electropherograms were analyzed using GeneMapper v. 5 (Applied Biosystems).

To study genetic diversity in polyploid *C. integerrimus* populations, we determined the multilocus genetic genotypes (MLGs or simply genotypes) for each individual on the basis of microsatellite allele data for each of five loci in the software program GenoType v. 1.2 [82]. Assignment of individuals into a particular clone was performed using the Meirmans and Van Tienderen [82] algorithm according to the calculation of a genetic distance matrix and a clonal threshold (set to two for the study, after testing different threshold values upon recommendations by the authors of the programs) under the stepwise mutation model option. In order to analyze clonal diversity of *C. integerrimus*, we included following measures: the total number of genotypes (Ng) and effective genotypes (Eff), the number of unique genotypes (Un), and Simpson’s diversity index (D) calculated in the software GenoDive v. 1.2 [82]. The proportion of distinguishable genotypes (Ng⁄ N) [83] was calculated as well. Relationships among multilocus genotypes were visualized by principal coordinate analysis (PCoA) based on Jaccard distances using PAST 3.17 [73].

The maximum number of alleles per loci corresponded to ploidy levels obtained using cytometry. For 65 individuals with unknown genome size, these data were used to infer the ploidy level (Table 2 and Table S2).

## 5. Conclusions

From genome size, ploidy level, mode of reproduction, and genotypic variation, we demonstrated that South European *C. integerrimus* populations from the Balkans represent a dynamic polyploid aggregate with complex breeding. The prevalence of apomictic tetraploids determines the overall cytotype structure of populations, confirming the pattern of geographic parthenogenesis along the sampled area. Genotypic variability of populations mainly results from the interaction of predominant facultative apomixis and residual sexual reproduction in tetraploids on a large geographical scale. Sexual diploids, due to their rarity, also contribute to the cytotype, reproductive diversity, and genotypic diversity of polyploid populations but at the local scale. The endosperm imbalance facilitates the development and the occurrence of intermediate triploids in mixed-ploidy populations, but also different tetraploid lineages elsewhere with unbalanced endosperm. South European populations of *C. integerrimus* showed higher levels of cytotype and reproductive diversity compared to the Central European ones, representing a potential reservoir of regional and global diversity for the species. Future efforts should be focused on more extensive sampling strategy of *C. integerrimus* populations to reveal the underlying processes shaping the diversity of this multiploid species.

## Figures and Tables

**Table 1:** Absolute genome size values and ploidy levels of *Cotoneaster integerrimus* cytotypes.

DNA Ploidy	*N*	2C DNA		CV %	1Cx DNA	
		Mean ± SD (pg)	Min–Max (pg)		Mean ± SD (pg) *	Mean (Mbp **)
2x	20	1.280 ± 0.046	1.23–1.36	2.47	0.636 ± 0.015 ^a^	622.00
3x	7	1.856 ± 0.091	1.63–1.90	5.04	0.608 ± 0.030 ^b^	594.62
4x	181	2.481 ± 0.060	2.36–2.73	2.45	0.621 ± 0.015 ^b^	607.73
TOTAL	208					

* Mean ± SD (pg) followed by the same letter in superscript was not significantly different according to the Tukey’s HSD test; ** 1pg = 978 Mbp [41].

**Table 2:** Geographic origin of *Cotoneaster* mother individuals covering three cytotypes and corresponding sample sizes (*N*) for genome size/ploidy level, reproductive mode, and microsatellite analysis.

		Origin of Sample				Number of Samples (*N*)			
Site No.	Site ID	Locality	East	North	Altitude (m)	Diploids	Triploids	Tetraploids	Total *N* per Site
1	Go	Gornja Grkarica, Mt. Igman, Bosnia and Herzegovina (B&H)	18.28833	43.74528	1350	-, -, -	-, -, -	20, 98, 19	20, 98, 19
2	Um	Umoljani, Mt. Bjelašnica, B&H	18.22167	43.66194	1300	18, 108, 12	4, 3, 4	8, 27, 8	30, 138, 24
3	Vr	Vrbe, Čemerno, B&H	18.57167	43.17861	1090	-, -, -	1, -, -	19, -, 18	20, -, 18
4	Bo	Bosiljna, Mt. Čvrsnica, B&H	17.48806	43.51194	1319	-, -, -	-, -, -	20, 7, 16	20, 7, 16
5	Ru	Rujište, Mt. Prenj, B&H	17.95472	43.46194	1010	-, -, -	1, -, 1	17, 14, 18	18,14, 19
6	Bg	Borova glava, Livno, B&H	17.12139	43.77861	1183	1, 3, -	-, -, -	22, 28, 21	23, 31, 21
7	So	Sovićka vrata, Blidinje, B&H	17.50472	43.59528	1220	-, -, -	1, -, 1	16, 4, 13	17, 4, 14
8	Pu	Puhova ravan, Mt. Trebević, B&H	18.43833	43.82861	1450	-, -, -	-, -, -	20, 55, 20	20, 55, 20
9	Ca	Čavljak, Mt. Ozren, B&H	18.43833	43.91194	1237	-, -, -	-, -, -	21, 75, 19	21, 75, 19
10	De	Devečanske stijene, Mt. Vlašić, B&H	17.62139	44.27889	1752	1, 2, 1	-, -, -	18, 57, 18	19, 59, 19
11	Rt	Mt. Rtanj, Serbia	21.88917	43.77861	1282	-, -, -	1 *, -, 1	8 *, -, 8	9, -, 9
12	Mu	Mt. Mučanj, Serbia	20.03889	43.54528	1530	-, -, -	-, -, -	7 *, 36, 7	7, 36, 7
13	Pr	Premužićeva staza, Mt. Velebit, Croatia	14.98722	44.76222	1613	-, -, -	1 *, -, 1	3 *, 2, 3	4, 2, 4
14	Vo	Vošac, Mt. Biokovo, Croatia	17.05472	43.31194	1320	-, -, -	1 *, -, 1	8 *, 58, 8	9, 58, 9
15	Si	Mt. Sinjajevina, Monte Negro	19.33861	42.96167	1773	-, -, -	-, -, -	5 *, -, 5	5, -, 5
16	He	Hechenberg-Ostgipfel von Kranebitten, Innsbruck, Austria	11.31972	47.27972	1390	-, -, -	-, -, -	5 *, -, 5	5, -, 5
17	Ro	Rotenfels, Rheinland-Pfalz, Germany	7.818611	49.83028	232	-, -, -	-, -, -	5 *, 6, 5	5, 6, 5
18	Mo	Monte Ventosola, Monte Sibillini, Umbria, Italy	13.17028	42.77833	1493	-, -, -	-, -, -	4 *, 8, 4	4, 8, 4
19	Su	Mt. Suva Gora, Northern Macedonia	21.25583	41.84472	1184	-, -, -	-, -, -	7 *, -, 7	7, -, 7
20	Ol	Mt. Olympus, Greece	22.37278	40.07778	1935	-, -, -	-, -, -	5 *, -, 5	5, -, 5
21	Pa	Mt. Pangaion, Greece	24.07333	40.92778	1662	-, -, -	-, -, -	5 *, -, 5	5, -, 5
		Total *N* per cytotype				20, 113, 13	10, 3, 9	243, 475, 232	273, 591, 254

* Ploidy level was inferred from microsatellite data.

**Table 3:** Flow cytometric seed screening results and hypothesized pathways of seed formation and reproduction modes in analyzed *Cotoneaster integerrimus* populations.

Cytometric Results						Hypothesized Pathways of Seed Formation						
Maternal ploidy ^a^	Embryopg ± SD	Endosperm pg ± SD	Embryo ploidy	Endosperm ploidy	Number of seeds	Egg ploidy	Polar nuclei ploidy/number	Sperm ploidy	Number of sperms fecundating the endosperm	Endosperm maternal: paternal genome ratio	Endosperm/embryo fluorescence (mean ratio)	Seed origin
2x	1.28 ± 0.03	1.95 ± 0.05	2x	3x	103	1x	1x/2	1x	1	2m:1p	1.52	Sexual
2x	1.91 ± 0.05	2.59 ± 0.06	3x	4x	8 ^b^	1x	1x/2	2x	1	1m:1p	1.35	Sexual
3x	1.94	2.64	3x	4x	1	1x	1x/2	2x	1	1m:1p	1.5	Sexual
3x	1.85	3.93	3x	7x	1	3x	3x/2	1x	1	6m:1p	1.35	Pseudogamous Apomixis
3x	1.94	5.72	3x	9x	1	3x	3x/2	3x	1	2m:1p	2.12	Pseudogamous Apomixis
4x	2.54 ± 0.07	3.84 ± 0.11	4x	6x	92	2x	2x/2	2x	1	2m:1p	1.53	Sexual
4x	3.80 ± 0.10	6.43 ± 0.21	6x	10x	22 ^c^	4x	4x/2	2x	2	4m:1p	2.5	Sexual
4x	1.90 ± 0.01	3.19 ± 0.05	3x	5x	2 ^d^	2x	2x/1	1x	1	4m:1p	1.7	Sexual
4x	2.56 ± 0.1	6.38 ± 0.15	4x	10x	148	4x	4x/2	1x or 2x	2 or 1	4m:1p	2.5	Pseudogamous Apomixis
4*x*	2.54 ± 0.01	7.51 ± 0.37	4x	12x	137	4x	4x/2	2x	2	2m:1p	2.98	Pseudogamous Apomixis
4x	2.55 ± 0.04	7.04 ± 0.25	4x	11x	22	4x	4x/2	3x	1	2.7m:1p	2.75	Pseudogamous Apomixis
4x	2.58 ± 0.12	5.83 ± 0.28	4x	9x	11	4x	4x/2	1x	1	8m:1p	2.17	Pseudogamous Apomixis
4x	2.53 ± 0.05	5.08 ± 0.17	4x	8x	32	4x	4x/2	-	0	4m:0p	4:1	Autonomous Apomixis
4x	2.55 ± 0.08	4.46 ± 0.17	4x	7x	9	4x						Not resolved
4x	1.28 ± 0.00	3.84 ± 0.04	2x	6x	2	2x	2x/2	1x or 2x	2 or 1			Haploid Parthenogenesis
					591							

^a^ Maternal ploidy inferred from cytometric leaf measurements. ^b^ Embryo ploidy was increased compared to mother ploidy. ^c^ Biii hybrids (explanation Section 2.2.3). ^d^ Embryo ploidy was decreased compared to mother ploidy.

**Table 4:** Genetic diversity measures for the analyzed *Cotoneaster integerrimus* populations derived from nuclear microsatellites.

Locality (Site ID)	Number of Individuals Sampled (*N*)	Number of Multilocus Genotypes Detected (*Ng*)	Total Number of Individuals Belonging to a Clone (*Ni*)	Total Number of Unique Genotypes per Population (*Un*)	Effective Number of Genotypes (*Eff*)	Proportion of Clones Detected (*Ng/N*)	Genotypic Diversity (*Div*)
Gornja Grkarica (Go)	19	3	18	1	2.21	0.15	0.57
Umoljani (Um)	24	21	4	19	18	0.91	0.98
Vrbe (Vr)	18	7	12	6	4.37	0.39	0.81
Bosiljna (Bo)	16	5	14	5	3.55	0.31	0.76
Rujište (Ru)	19	4	17	4	2.39	0.21	0.61
Borova glava (Bg)	21	2	20	1	1.09	0.1	0.09
Sovićka vrata (So)	14	6	11	4	4.07	0.42	0.81
Puhova ravan (Pu)	20	1	20	1	1	0.05	0
Čavljak (Ca)	19	5	16	4	1.57	0.26	0.38
Devečanske stijene (De)	19	6	16	6	2.63	0.31	0.65
Mt. Rtanj (Rt)	9	4	7	4	3	0.44	0.75
Mt. Mučanj (Mu)	7	2	7	2	1.86	0.28	0.47
Premužićeva staza (Pr)	4	3	2	3	2.66	1	0.83
Vošac (Vo)	9	4	7	3	2.07	0.33	0.58
Mt. Sinjajevina (Si)	5	1	5	1	1	0.20	0
Hechenberg-Ostgipfel (He)	5	1	5	1	1	0.20	0
Rotenfels (Ro)	5	3	3	3	2.27	0.60	0.70
Monte Ventosola (Mo)	4	3	2	3	2.66	0.75	0.83
Mt. Suva Gora (Su)	7	3	4	2	1.81	0.71	0.52
Mt. Olympus (Ol)	5	4	2	4	3.57	0.8	0.90
Mt. Pangaion (Pa)	5	4	2	4	3.57	0.8	0.90
	254	92	194	81			

## Data Availability

Not applicable.

## Supplementary Materials

The following are available online at https://www.mdpi.com/article/10.3390/plants10122798/s1, Table S1: Individual FCSS profiles. Table S2: Genome composition of the studied *Cotoneaster integerrimus* individuals at five microsatellite loci. Figure S1: Principal coordinate analysis of multilocus genotypes from the Umoljani site.
