# Supplementary material for: Genome Size, Cytotype Diversity and Reproductive Mode Variation of Cotoneaster integerrimus (Rosaceae) from the Balkans
Source: Plants (Basel). 2021 Dec 17;10(12):2798. doi: 10.3390/plants10122798 (PMC8708406; doi:10.3390/plants10122798)
Supplement: Supplementary file 1 [file plants-10-02798-s001.zip › Table S1. FCSS profiles_Bogunic et al_2021_Plants.pdf]

Table S1. Flow cytometric seed screening results for *Cotoneaster integerrimus* populations.

Site	Site ID_Mother number_Seed number	Mother ploidy	Genome size of embryo (pg)	Ratio of endosperm and standard fluorescence	Genome size of endosperm (pg)	Embryo ploidy	Endosperm ploidy
**Borova glava**	Bg_13_9	4x	2,54	3,84	3,76	4x	6x
	Bg_13_10	4x	3,62	6,24	6,12	6x	10x
	Bg_13_11	4x	2,41	7,64	7,48	4x	12x
	Bg_13_12	4x	2,50	6,41	6,28	4x	10x
	Bg_13_13	4x	2,51	7,74	7,59	4x	12x
	Bg_13_14	4x	2,45	6,43	6,30	4x	10x
	Bg_13_15	4x	2,58	6,32	6,19	4x	10x
	Bg_13_17	4x	2,42	3,70	3,63	4x	6x
	Bg_13_18	4x	2,51	4,76	4,67	4x	7x
	Bg_13_19	4x	2,48	7,74	7,59	4x	12x
	Bg_13_20	4x	2,68	8,05	7,89	4x	12x
	Bg_13_21	4x	2,62	7,95	7,79	4x	12x
	Bg_13_22	4x	2,41	4,64	4,54	4x	8x
	Bg_13_23	4x	2,55	7,84	7,68	4x	12x
	Bg_15_1	4x	2,54	6,42	6,29	4x	10x
	Bg_15_2	4x	2,51	6,51	6,38	4x	10x
	Bg_15_3	4x	3,92	6,68	6,55	6x	10x
	Bg_15_4	4x	2,64	8,01	7,85	4x	12x
	Bg_15_5	4x	2,62	6,68	6,55	4x	10x
	Bg_15_6	4x	2,51	6,60	6,47	4x	10x
	Bg_15_7	4x	2,54	7,86	7,70	4x	12x
	Bg_15_8	4x	2,61	7,84	7,69	4x	12x
	Bg_15_9	4x	2,48	7,09	6,95	4x	11x
	Bg_6_1A	4x	2,44	3,95	3,87	4x	6x
	Bg_6_1	4x	2,61	5,03	4,93	4x	8x
	Bg_6_2	4x	2,51	3,95	3,87	4x	6x
	Bg_6_3	4x	2,61	7,83	7,67	4x	12x
	Bg_6_4	4x	2,63	3,90	3,83	4x	6x
	Bg_7_1	2x	1,23	1,99	1,95	2x	3x
	Bg_7_2	2x	1,28	1,98	1,94	2x	3x
	Bg_7_3	2x	1,27	1,99	1,95	2x	3x
	Bg_8_4	4x	2,55	6,40	6,27	4x	10x
	Bg_8_4	4x	2,64	4,05	3,97	4x	6x
**Bosiljna**	Bo_3_2	4x	2,55	7,74	7,59	4x	12x
	Bo_3_3	4x	2,58	7,95	7,79	4x	12x
	Bo_3_5	4x	2,51	7,74	7,59	4x	12x
	Bo_3_6	4x	2,58	7,84	7,69	4x	12x
	Bo_3_8	4x	2,54	7,85	7,69	4x	12x
	Bo_8_1	4x	2,58	6,50	6,37	4x	10x
	Bo_8_2	4x	2,58	7,74	7,58	4x	12x
**Čavljak**	Ca_11_1	4x	2,54	7,84	7,69	4x	12x
	Ca_11_2	4x	2,57	6,63	6,49	4x	10x
	Ca_11_3	4x	2,57	7,14	7,00	4x	11x
	Ca_11_5	4x	2,56	6,61	6,48	4x	10x
	Ca_14_1	4x	2,58	7,64	7,49	4x	12x
	Ca_14_2	4x	2,58	7,74	7,59	4x	12x
	Ca_15_1	4x	2,72	7,98	7,82	4x	12x
	Ca_15_10	4x	2,45	7,54	7,39	4x	12x
	Ca_15_11	4x	2,48	7,54	7,39	4x	12x
	Ca_15_12	4x	2,55	7,84	7,68	4x	12x
	Ca_15_13	4x	2,57	4,00	3,92	4x	6x
	Ca_15_14	4x	2,51	6,32	6,20	4x	10x

Ca_15_15	4x	2,48	6,29	6,17	4x	10x
Ca_15_16	4x	2,58	6,69	6,56	4x	10x
Ca_15_17	4x	2,48	6,32	6,19	4x	10x
Ca_15_18	4x	2,55	7,84	7,68	4x	12x
Ca_15_19	4x	2,48	7,64	7,48	4x	12x
Ca_15_2	4x	2,41	7,43	7,29	4x	12x
Ca_15_20	4x	2,44	7,43	7,29	4x	12x
Ca_15_21	4x	2,48	7,63	7,48	4x	12x
Ca_15_22	4x	2,54	7,85	7,69	4x	12x
Ca_15_23	4x	2,54	7,74	7,58	4x	12x
Ca_15_24	4x	2,51	7,74	7,59	4x	12x
Ca_15_25	4x	2,54	7,85	7,69	4x	12x
Ca_15_26	4x	2,50	7,74	7,59	4x	12x
Ca_15_27	4x	2,44	7,43	7,28	4x	12x
Ca_15_29	4x	2,37	7,24	7,09	4x	12x
Ca_15_3	4x	2,51	7,74	7,58	4x	12x
Ca_15_4	4x	2,45	6,43	6,30	4x	10x
Ca_15_5	4x	2,49	7,66	7,51	4x	12x
Ca_15_6	4x	2,48	6,33	6,21	4x	10x
Ca_15_7	4x	2,55	7,86	7,70	4x	12x
Ca_15_8	4x	2,51	7,64	7,49	4x	12x
Ca_15_9	4x	2,48	7,65	7,50	4x	12x
Ca_16_1	4x	2,50	7,60	7,45	4x	12x
Ca_16_10	4x	2,57	7,80	7,64	4x	12x
Ca_16_11	4x	2,50	4,04	3,95	4x	6x
Ca_16_12	4x	2,57	4,08	4,00	4x	6x
Ca_16_13	4x	2,51	5,09	4,99	4x	8x
Ca_16_14	4x	2,46	4,02	3,94	4x	6x
Ca_16_15	4x	2,57	7,80	7,64	4x	12x
Ca_16_16	4x	2,65	7,95	7,79	4x	12x
Ca_16_17	4x	2,53	3,87	3,79	4x	6x
Ca_16_19	4x	2,55	6,52	6,39	4x	10x
Ca_16_2	4x	2,61	7,40	7,25	4x	11x
Ca_16_20	4x	2,60	7,48	7,33	4x	11x
Ca_16_21	4x	2,42	6,46	6,33	4x	10x
Ca_16_22	4x	2,50	7,51	7,36	4x	12x
Ca_16_3	4x	2,55	7,65	7,49	4x	12x
Ca_16_4	4x	2,55	7,33	7,19	4x	11x
Ca_16_5	4x	2,48	7,53	7,38	4x	12x
Ca_16_6	4x	2,61	6,74	6,61	4x	10x
Ca_16_7	4x	2,57	7,31	7,16	4x	11x
Ca_16_8	4x	2,53	7,60	7,44	4x	12x
Ca_16_9	4x	2,48	7,53	7,38	4x	12x
Ca_17_1	4x	2,55	7,67	7,51	4x	12x
Ca_17_10	4x	2,55	7,70	7,55	4x	12x
Ca_17_11	4x	2,55	6,60	6,47	4x	10x
Ca_17_12	4x	3,77	6,51	6,38	6x	10x
Ca_17_13	4x	2,62	7,86	7,70	4x	12x
Ca_17_14	4x	2,53	7,69	7,53	4x	12x
Ca_17_2	4x	2,48	7,88	7,72	4x	12x
Ca_17_3	4x	2,47	7,51	7,36	4x	12x
Ca_17_4	4x	2,66	8,08	7,91	4x	12x
Ca_17_5	4x	2,48	6,52	6,39	4x	10x
Ca_17_6	4x	2,48	7,46	7,31	4x	12x
Ca_17_7	4x	2,52	7,57	7,42	4x	12x
Ca_17_8	4x	2,48	4,53	4,44	4x	7x
Ca_17_9	4x	2,52	3,91	3,84	4x	6x
Ca_2_1	4x	2,45	3,79	3,72	4x	6x
Ca_8_1	4x	2,51	7,66	7,51	4x	12x
Ca_8_2	4x	2,48	7,44	7,29	4x	12x
Ca_8_3	4x	2,52	6,42	6,29	4x	10x
Ca_8_4	4x	2,62	4,00	3,92	4x	6x

	Ca_8_5	4x	2,49	6,45	6,32	4x	10x
**Rotenfels**	Ro_1_1	4x	2,55	5,12	5,02	4x	8x
	Ro_1_2	4x	2,51	7,69	7,54	4x	12x
	Ro_1_3	4x	3,77	6,42	6,29	6x	10x
	Ro_1_4	4x	2,58	7,54	7,39	4x	12x
	Ro_1_5	4x	2,51	6,50	6,37	4x	10x
	Ro_1_6	4x	2,54	6,58	6,45	4x	10x
**Gornja grkarica**	Go_10_1	4x	2,66	6,87	6,73	4x	10x
	Go_10_10	4x	3,71	6,32	6,20	6x	10x
	Go_10_11	4x	2,50	6,51	6,38	4x	10x
	Go_10_12	4x	2,55	3,96	3,88	4x	6x
	Go_10_13	4x	2,58	3,94	3,86	4x	6x
	Go_10_14	4x	2,50	6,40	6,27	4x	10x
	Go_10_15	4x	2,48	3,84	3,77	4x	6x
	Go_10_16	4x	2,55	5,37	5,27	4x	8x
	Go_10_17	4x	2,62	6,76	6,63	4x	10x
	Go_10_18	4x	2,55	5,40	5,29	4x	8x
	Go_10_19	4x	2,59	4,07	3,99	4x	6x
	Go_10_2	4x	2,58	6,68	6,55	4x	10x
	Go_10_20	4x	2,53	6,59	6,46	4x	10x
	Go_10_21	4x	2,51	6,52	6,39	4x	10x
	Go_10_22	4x	2,58	4,06	3,98	4x	6x
	Go_10_23	4x	2,55	6,62	6,49	4x	10x
	Go_10_26	4x	2,48	6,50	6,37	4x	10x
	Go_10_27	4x	2,54	4,00	3,92	4x	6x
	Go_10_28	4x	2,58	6,69	6,56	4x	10x
	Go_10_29	4x	2,59	6,79	6,66	4x	10x
	Go_10_3	4x	2,65	6,73	6,60	4x	10x
	Go_10_30	4x	2,54	6,67	6,53	4x	10x
	Go_10_31	4x	2,59	7,27	7,12	4x	11x
	Go_10_32	4x	2,51	3,84	3,76	4x	6x
	Go_10_4	4x	2,68	6,59	6,46	4x	10x
	Go_10_5	4x	2,58	4,00	3,92	4x	6x
	Go_10_6	4x	2,58	6,71	6,58	4x	10x
	Go_10_7	4x	2,58	6,59	6,46	4x	10x
	Go_10_8	4x	2,40	3,68	3,61	4x	6x
	Go_10_9	4x	2,55	3,90	3,82	4x	6x
	Go_11_1	4x	2,54	4,23	4,14	4x	7x
	Go_11_10	4x	2,58	6,48	6,35	4x	10x
	Go_11_11	4x	2,48	6,32	6,20	4x	10x
	Go_11_13	4x	2,58	5,17	5,07	4x	8x
	Go_11_14	4x	3,75	6,56	6,43	6x	10x
	Go_11_15	4x	2,54	3,84	3,76	4x	6x
	Go_11_16	4x	2,61	6,59	6,46	4x	10x
	Go_11_17	4x	2,50	6,32	6,19	4x	10x
	Go_11_18	4x	2,58	5,76	5,65	4x	9x
	Go_11_19	4x	2,57	4,00	3,92	4x	6x
	Go_11_2	4x	2,58	6,15	6,03	4x	9x
	Go_11_20	4x	2,53	7,66	7,51	4x	12x
	Go_11_21	4x	2,51	7,69	7,54	4x	12x
	Go_11_22	4x	2,52	3,85	3,78	4x	6x
	Go_11_23	4x	2,61	4,06	3,98	4x	6x
	Go_11_24	4x	2,61	5,17	5,07	4x	8x
	Go_11_25	4x	2,38	3,74	3,67	4x	6x
	Go_11_3	4x	2,50	3,83	3,76	4x	6x
	Go_11_5	4x	2,70	4,08	3,99	4x	6x
	Go_11_6	4x	2,51	3,90	3,82	4x	6x
	Go_11_7	4x	2,55	3,89	3,81	4x	6x
	Go_11_8	4x	2,54	5,16	5,06	4x	8x
	Go_11_9	4x	2,58	6,59	6,46	4x	10x
	Go_14_1	4x	2,58	3,94	3,87	4x	6x

	Go_14_10	4x	2,54	3,95	3,87	4x	6x
	Go_14_3	4x	2,58	5,10	5,00	4x	8x
	Go_14_4	4x	2,54	4,11	4,03	4x	6x
	Go_14_5	4x	2,55	5,31	5,20	4x	8x
	Go_14_6	4x	2,58	4,40	4,31	4x	7x
	Go_14_7	4x	2,54	4,96	4,86	4x	8x
	Go_14_8	4x	2,54	4,51	4,42	4x	7x
	Go_14_9	4x	2,51	4,57	4,48	4x	7x
	Go_15_1	4x	2,61	6,67	6,53	4x	10x
	Go_15_10	4x	2,62	6,57	6,44	4x	10x
	Go_15_11	4x	2,61	5,32	5,21	4x	8x
	Go_15_12	4x	2,63	3,90	3,83	4x	6x
	Go_15_13	4x	2,58	6,67	6,53	4x	10x
	Go_15_15	4x	2,55	5,25	5,15	4x	8x
	Go_15_16	4x	2,58	6,87	6,73	4x	10x
	Go_15_17	4x	2,58	3,90	3,82	4x	6x
	Go_15_18	4x	2,62	6,69	6,56	4x	10x
	Go_15_19	4x	2,62	6,70	6,57	4x	10x
	Go_15_2	4x	2,63	6,97	6,83	4x	10x
	Go_15_20	4x	2,55	3,90	3,82	4x	6x
	Go_15_22	4x	2,62	4,06	3,98	4x	6x
	Go_15_23	4x	2,63	4,08	3,99	4x	6x
	Go_15_24	4x	2,55	5,57	5,46	4x	9x
	Go_15_25	4x	2,58	6,69	6,56	4x	10x
	Go_15_26	4x	2,49	3,74	3,66	4x	6x
	Go_15_27	4x	2,59	4,01	3,93	4x	6x
	Go_15_29	4x	2,45	3,79	3,72	4x	6x
	Go_15_3	4x	2,78	5,22	5,11	4x	7x
	Go_15_30	4x	2,59	6,61	6,48	4x	10x
	Go_15_4	4x	2,58	6,58	6,44	4x	10x
	Go_15_5	4x	2,54	3,95	3,87	4x	6x
	Go_15_6	4x	2,58	6,76	6,63	4x	10x
	Go_15_7	4x	2,58	6,48	6,35	4x	10x
	Go_15_8	4x	2,44	3,74	3,66	4x	6x
	Go_15_9	4x	2,67	6,97	6,83	4x	10x
	Go_20_1	4x	2,57	5,31	5,20	4x	8x
	Go_20_2	4x	2,50	5,10	5,00	4x	8x
	Go_20_3	4x	2,55	5,39	5,28	4x	8x
	Go_20_4	4x	2,62	3,94	3,86	4x	6x
	Go_20_5	4x	2,58	3,89	3,81	4x	6x
	Go_20_6	4x	2,55	6,41	6,29	4x	10x
	Go_20_7	4x	2,54	5,09	4,99	4x	8x
	Go_20_8	4x	2,57	6,40	6,27	4x	10x
**Monte Ventosola**	Mo_1	4x	2,73	6,59	6,46	4x	9x
	Mo_1_7	4x	2,57	4,00	3,92	4x	6x
	Mo_1_8	4x	2,54	3,95	3,87	4x	6x
	Mo_2	4x	2,58	6,58	6,45	4x	10x
	Mo_3	4x	2,86	6,37	6,24	4x	9x
	Mo_4	4x	2,67	6,86	6,72	4x	10x
	Mo_5	4x	3,77	6,60	6,47	6x	10x
	Mo_6	4x	2,51	6,41	6,28	4x	10x
**Mučanj**	Mu_1_1	4x	2,57	7,73	7,58	4x	12x
	Mu_1_2	4x	2,58	7,74	7,58	4x	12x
	Mu_1_3	4x	2,58	6,58	6,45	4x	10x
	Mu_1_4	4x	2,58	7,96	7,80	4x	12x
	Mu_1_5	4x	2,58	7,96	7,80	4x	12x
	Mu_1_7	4x	2,58	6,59	6,46	4x	10x
	Mu_1_8	4x	2,50	6,40	6,27	4x	10x
	Mu_4_10	4x	2,43	5,05	4,95	4x	8x
	Mu_4_11	4x	2,52	3,90	3,82	4x	6x
	Mu_4_13	4x	2,48	4,97	4,87	4x	8x

	Mu_4_14	4x	2,55	5,38	5,27	4x	8x
	Mu_4_15	4x	2,50	5,96	5,85	4x	9x
	Mu_4_16	4x	2,57	6,55	6,42	4x	10x
	Mu_4_17	4x	2,55	6,40	6,27	4x	10x
	Mu_4_18	4x	2,48	7,43	7,28	4x	12x
	Mu_4_19	4x	2,56	6,51	6,38	4x	10x
	Mu_4_2	4x	2,54	4,51	4,42	4x	7x
	Mu_4_20	4x	2,54	7,55	7,40	4x	12x
	Mu_4_21	4x	3,64	6,21	6,09	6x	10x
	Mu_4_22	4x	2,37	7,27	7,13	4x	12x
	Mu_4_23	4x	2,66	6,87	6,73	4x	10x
	Mu_4_24	4x	2,53	7,69	7,53	4x	12x
	Mu_4_25	4x	2,53	6,45	6,32	4x	10x
	Mu_4_26	4x	3,69	6,29	6,16	6x	10x
	Mu_4_27	4x	2,53	3,87	3,79	4x	6x
	Mu_4_29	4x	2,55	6,35	6,22	4x	10x
	Mu_4_3	4x	2,55	6,49	6,36	4x	10x
	Mu_4_30	4x	3,73	6,45	6,32	6x	10x
	Mu_4_5	4x	2,55	3,87	3,79	4x	6x
	Mu_4_6	4x	2,48	6,67	6,53	4x	11x
	Mu_4_7	4x	2,49	5,11	5,01	4x	8x
	Mu_4_8	4x	2,52	5,24	5,13	4x	8x
	Mu_4_9	4x	2,57	5,42	5,32	4x	8x
	Mu_7_1	4x	2,54	7,74	7,58	4x	12x
	Mu_7_2	4x	2,58	6,58	6,45	4x	10x
	Mu_7_3	4x	2,58	7,95	7,79	4x	12x
**Rujište**	Ru_1_1	4x	2,51	6,60	6,47	4x	10x
	Ru_2_1	4x	3,82	6,59	6,46	6x	10x
	Ru_2_2	4x	2,61	4,00	3,92	4x	6x
	Ru_3_1	4x	2,58	6,50	6,37	4x	10x
	Ru_3_2	4x	2,54	6,42	6,29	4x	10x
	Ru_3_3	4x	2,61	7,35	7,20	4x	11x
	Ru_3_4	4x	2,58	6,68	6,55	4x	10x
	Ru_3_5	4x	2,55	7,35	7,20	4x	11x
	Ru_3_6	4x	2,58	7,44	7,29	4x	11x
	Ru_A_1	4x	2,58	3,90	3,82	4x	6x
	Ru_B_1	4x	2,45	3,85	3,77	4x	6x
	Ru_R_1	4x	2,48	3,85	3,77	4x	6x
	Ru_R_2	4x	2,58	7,14	7,00	4x	11x
	Ru_R_3	4x	2,54	7,44	7,29	4x	12x
	Ru_R_5	4x	2,55	6,52	6,39	4x	10x
	Ru_R_6	4x	2,58	7,44	7,29	4x	11x
**Sovićka vrata**	So_11_9_10_11	4x	2,51	3,79	3,72	4x	6x
	So_14_1	2x	1,22	1,86	1,82	2x	3x
**Puhova ravan**	Pu_1_1	4x	3,82	6,51	6,38	6x	10x
	Pu_1_2	4x	2,54	7,65	7,49	4x	12x
	Pu_1_3	4x	2,56	7,57	7,42	4x	12x
	Pu_1_4	4x	2,52	6,35	6,23	4x	10x
	Pu_10_1	4x	2,55	6,78	6,64	4x	10x
	Pu_10_2	4x	2,34	3,48	3,41	4x	6x
	Pu_10_3	4x	2,56	7,69	7,53	4x	12x
	Pu_10_4	4x	2,50	3,92	3,84	4x	6x
	Pu_10_5	4x	3,68	6,36	6,23	6x	10x
	Pu_10_6	4x	2,55	3,85	3,77	4x	6x
	Pu_10_7	4x	3,68	6,53	6,40	6x	10x
	Pu_10_8	4x	2,47	7,21	7,07	4x	11x
	Pu_10_9	4x	2,59	3,90	3,82	4x	6x
	Pu_14_1	4x	2,54	7,06	6,92	4x	11x
	Pu_14_10	4x	2,45	5,12	5,02	4x	8x
	Pu_14_11	4x	3,87	5,92	5,81	4x	6x
	Pu_14_12	4x	2,51	3,90	3,82	4x	6x

	Pu_14_13	4x	2,51	6,59	6,46	4x	10x
	Pu_14_14	4x	2,60	6,71	6,58	4x	10x
	Pu_14_15	4x	2,63	6,62	6,49	4x	10x
	Pu_14_2	4x	3,77	6,50	6,37	6x	10x
	Pu_14_3	4x	2,59	4,01	3,93	4x	6x
	Pu_14_5	4x	2,51	6,59	6,45	4x	10x
	Pu_14_6	4x	2,58	6,87	6,73	4x	10x
	Pu_14_8	4x	2,59	7,27	7,12	4x	11x
	Pu_14_9	4x	2,59	6,60	6,47	4x	10x
	Pu_4_1	4x	2,49	7,98	7,82	4x	12x
	Pu_4_10	4x	2,55	7,84	7,68	4x	12x
	Pu_4_11	4x	2,42	7,28	7,13	4x	12x
	Pu_4_2	4x	2,48	3,85	3,77	4x	6x
	Pu_4_3	4x	2,52	6,61	6,48	4x	10x
	Pu_4_4	4x	2,48	6,35	6,22	4x	10x
	Pu_4_5	4x	2,52	7,57	7,42	4x	12x
	Pu_4_6	4x	2,52	7,67	7,51	4x	12x
	Pu_4_7	4x	2,63	4,08	3,99	4x	6x
	Pu_4_8	4x	2,45	6,44	6,31	4x	10x
	Pu_4_9	4x	2,53	6,36	6,23	4x	10x
	Pu_8_1	4x	2,48	6,76	6,63	4x	11x
	Pu_8_10	4x	2,48	5,19	5,08	4x	8x
	Pu_8_12	4x	2,51	7,74	7,59	4x	12x
	Pu_8_13	4x	2,51	3,84	3,77	4x	6x
	Pu_8_14	4x	2,51	7,74	7,58	4x	12x
	Pu_8_15	4x	2,51	6,51	6,38	4x	10x
	Pu_8_16	4x	2,48	5,68	5,57	4x	9x
	Pu_8_17	4x	2,54	7,65	7,49	4x	12x
	Pu_8_18	4x	2,44	6,23	6,11	4x	10x
	Pu_8_19	4x	2,48	6,40	6,27	4x	10x
	Pu_8_2	4x	2,58	7,96	7,80	4x	12x
	Pu_8_3	4x	3,66	6,41	6,28	6x	10x
	Pu_8_4	4x	2,58	7,64	7,48	4x	12x
	Pu_8_5	4x	2,51	3,89	3,82	4x	6x
	Pu_8_6	4x	2,47	7,64	7,49	4x	12x
	Pu_8_7	4x	2,45	7,54	7,39	4x	12x
	Pu_8_8	4x	2,48	6,42	6,29	4x	10x
	Pu_8_9	4x	2,39	7,30	7,15	4x	12x
**Umoljani**	Um_11A_1	4x	2,55	6,35	6,22	4x	10x
	Um_11A_2	4x	2,55	5,46	5,35	4x	8x
	Um_11A_4	4x	2,56	4,91	4,82	4x	8x
	Um_12A_1	2x	1,36	2,12	2,08	2x	3x
	Um_12A_2	2x	1,36	2,06	2,02	2x	3x
	Um_12A_3	2x	2,01	2,76	2,70	3x	4x
	Um_12A_4	2x	1,54	2,42	2,37	2x	3x
	Um_12A_5	2x	1,36	2,10	2,06	2x	3x
	Um_13A_1	3x	1,95	5,83	5,72	3x	9x
	Um_13A_2	3x	1,85	4,01	3,93	3x	6x
	Um_1A_1	2x	1,26	1,95	1,91	2x	3x
	Um_1A_10	2x	1,27	1,96	1,93	2x	3x
	Um_1A_11	2x	1,27	1,95	1,91	2x	3x
	Um_1A_12	2x	1,25	1,93	1,89	2x	3x
	Um_1A_13	2x	1,30	1,97	1,93	2x	3x
	Um_1A_14	2x	1,30	1,97	1,93	2x	3x
	Um_1A_15	2x	1,29	1,98	1,94	2x	3x
	Um_1A_2	2x	1,26	1,97	1,93	2x	3x
	Um_1A_3	2x	1,26	1,98	1,94	2x	3x
	Um_1A_4	2x	1,29	1,99	1,95	2x	3x
	Um_1A_5	2x	1,27	1,96	1,93	2x	3x
	Um_1A_6	2x	1,26	1,97	1,93	2x	3x
	Um_1A_8	2x	1,33	2,05	2,01	2x	3x

Um_1A_9	2x	1,30	1,99	1,95	2x	3x
Um_1B_10	2x	1,28	1,99	1,95	2x	3x
Um_1B_11	2x	1,26	1,96	1,92	2x	3x
Um_1B_12	2x	1,28	2,01	1,97	2x	3x
Um_1B_13	2x	1,27	1,99	1,95	2x	3x
Um_1B_14	2x	1,28	1,99	1,95	2x	3x
Um_1B_15	2x	1,28	2,01	1,97	2x	3x
Um_1B_16	2x	1,24	1,96	1,92	2x	3x
Um_1B_17	2x	1,28	1,99	1,95	2x	3x
Um_1B_18	2x	1,87	2,59	2,54	3x	4x
Um_1B_19	2x	1,27	2,00	1,96	2x	3x
Um_1B_2	2x	1,25	2,01	1,97	2x	3x
Um_1B_20	2x	1,92	2,66	2,61	3x	4x
Um_1B_21	2x	1,27	1,96	1,92	2x	3x
Um_1B_22	2x	1,26	1,96	1,92	2x	3x
Um_1B_23	2x	1,27	1,99	1,95	2x	3x
Um_1B_24	2x	1,29	2,04	2,00	2x	3x
Um_1B_25	2x	1,27	2,01	1,97	2x	3x
Um_1B_3	2x	1,29	2,01	1,97	2x	3x
Um_1B_4	2x	1,29	2,07	2,03	2x	3x
Um_1B_5	2x	1,29	2,01	1,97	2x	3x
Um_1B_6	2x	1,25	1,99	1,95	2x	3x
Um_1B_7	2x	1,26	1,96	1,92	2x	3x
Um_1B_8	2x	1,26	1,93	1,89	2x	3x
Um_1B_9	2x	1,87	2,56	2,51	3x	4x
Um_2A_1	2x	1,31	2,04	2,00	2x	3x
Um_2A_2	2x	1,29	2,01	1,97	2x	3x
Um_2A_3	2x	1,97	2,71	2,65	3x	4x
Um_2A_4	2x	1,33	2,07	2,03	2x	3x
Um_2A_5	2x	1,34	2,04	2,00	2x	3x
Um_2B_1	2x	1,30	2,02	1,98	2x	3x
Um_2B_10	2x	1,27	1,94	1,90	2x	3x
Um_2B_2	2x	1,26	1,95	1,91	2x	3x
Um_2B_4	2x	1,29	1,97	1,93	2x	3x
Um_2B_5	2x	1,28	1,97	1,93	2x	3x
Um_2B_6	2x	1,30	2,00	1,96	2x	3x
Um_2B_7	2x	1,29	2,00	1,96	2x	3x
Um_2B_8	2x	1,29	2,00	1,96	2x	3x
Um_2B_9	2x	1,97	2,69	2,63	3x	4x
Um_3A_1	4x	1,92	3,26	3,19	3x	5x
Um_3A_1	4x	2,55	6,49	6,36	4x	10x
Um_3A_2	4x	2,58	6,41	6,28	4x	10x
Um_3B_1	2x	1,29	2,04	2,00	2x	3x
Um_3B_2	2x	1,26	1,99	1,95	2x	3x
Um_3B_3	2x	1,26	2,01	1,97	2x	3x
Um_3B_4	2x	1,26	1,99	1,95	2x	3x
Um_3B_5	2x	1,28	1,99	1,95	2x	3x
Um_3B_6	2x	1,29	2,04	2,00	2x	3x
Um_4A	3x	1,95	2,70	2,64	3x	4x
Um_4B_1	2x	1,28	1,99	1,95	2x	3x
Um_4B_2	2x	1,92	2,61	2,56	3x	4x
Um_4B_3	2x	1,27	1,98	1,94	2x	3x
Um_4B_4	2x	1,26	1,96	1,92	2x	3x
Um_4B_5	2x	1,27	1,97	1,93	2x	3x
Um_4B_6	2x	1,27	1,97	1,93	2x	3x
Um_4B_7	2x	1,24	1,93	1,90	2x	3x
Um_4B_8	2x	1,30	2,00	1,96	2x	3x
Um_5A_1	2x	1,28	1,97	1,93	2x	3x
Um_5A_2	2x	1,28	1,98	1,94	2x	3x
Um_5A_3	2x	1,31	1,99	1,95	2x	3x
Um_5A_4	2x	1,27	1,98	1,94	2x	3x

	Um_5A_5	2x	1,31	2,02	1,98	2x	3x
	Um_5A_6	2x	1,29	2,00	1,96	2x	3x
	Um_5A_7	2x	1,27	1,97	1,93	2x	3x
	Um_5B_1	4x	2,56	6,61	6,48	4x	10x
	Um_5B_10	4x	3,98	6,86	6,72	6x	10x
	Um_5B_11	4x	1,89	3,14	3,08	3x	5x
	Um_5B_2	4x	2,55	6,64	6,50	4x	10x
	Um_5B_3	4x	2,56	7,98	7,82	4x	12x
	Um_5B_4	4x	2,56	7,75	7,60	4x	12x
	Um_5B_5	4x	3,98	6,78	6,64	6x	10x
	Um_5B_6	4x	2,54	6,51	6,38	4x	10x
	Um_5B_7	4x	2,52	6,51	6,38	4x	10x
	Um_5B_8	4x	2,54	7,86	7,70	4x	12x
	Um_5B_9	4x	2,52	7,65	7,49	4x	12x
	Um_6A_10	4x	2,54	6,33	6,20	4x	10x
	Um_6A_11	4x	2,48	6,24	6,12	4x	10x
	Um_6A_11	4x	2,41	4,77	4,67	4x	8x
	Um_6A_2	4x	2,51	6,33	6,21	4x	10x
	Um_6A_3	4x	2,51	6,41	6,28	4x	10x
	Um_6A_4	4x	2,58	7,43	7,29	4x	11x
	Um_6A_5	4x	2,58	7,74	7,59	4x	12x
	Um_6A_6	4x	2,58	7,54	7,39	4x	12x
	Um_6A_7	4x	2,55	7,64	7,48	4x	12x
	Um_6A_8	4x	2,45	7,24	7,10	4x	12x
	Um_6A_9	4x	2,58	6,59	6,46	4x	10x
	Um_7A	2x	1,28	1,97	1,93	2x	3x
	Um_7B_1	2x	1,25	1,94	1,91	2x	3x
	Um_7B_2	2x	1,31	2,02	1,98	2x	3x
	Um_7B_3	2x	1,29	1,98	1,94	2x	3x
	Um_7B_4	2x	1,27	1,96	1,92	2x	3x
	Um_7B_5	2x	1,28	1,99	1,95	2x	3x
	Um_7B_6	2x	1,28	1,99	1,95	2x	3x
	Um_8B_1	2x	1,28	2,01	1,97	2x	3x
	Um_8B_10	2x	1,28	1,94	1,90	2x	3x
	Um_8B_11	2x	1,32	2,04	2,00	2x	3x
	Um_8B_12	2x	1,29	2,01	1,97	2x	3x
	Um_8B_13	2x	1,31	2,01	1,97	2x	3x
	Um_8B_14	2x	1,27	2,01	1,97	2x	3x
	Um_8B_15	2x	1,42	2,15	2,11	2x	3x
	Um_8B_16	2x	1,30	2,04	2,00	2x	3x
	Um_8B_17	2x	1,33	2,02	1,98	2x	3x
	Um_8B_18	2x	1,26	1,99	1,95	2x	3x
	Um_8B_19	2x	1,32	1,99	1,95	2x	3x
	Um_8B_2	2x	1,26	2,00	1,96	2x	3x
	Um_8B_20	2x	1,27	2,01	1,97	2x	3x
	Um_8B_21	2x	1,27	1,96	1,92	2x	3x
	Um_8B_22	2x	1,90	2,65	2,59	3x	4x
	Um_8B_23	2x	1,28	2,01	1,97	2x	3x
	Um_8B_24	2x	1,32	2,01	1,97	2x	3x
	Um_8B_3	2x	1,28	1,99	1,95	2x	3x
	Um_8B_4	2x	1,28	1,99	1,95	2x	3x
	Um_8B_5	2x	1,29	1,98	1,94	2x	3x
	Um_8B_6	2x	1,27	2,01	1,97	2x	3x
	Um_8B_8	2x	1,29	1,99	1,95	2x	3x
	Um_8B_9	2x	1,27	1,99	1,95	2x	3x
	Um_9A_1	2x	1,28	1,99	1,95	2x	3x
	Um_9A_2	2x	1,28	1,99	1,95	2x	3x
**Premužićeva staza**	Pr_4_1	4x	3,87	6,58	6,45	6x	10x
	Pr_4_3	4x	2,61	6,50	6,37	4x	10x
	Pr_10_1	4x	2,58	6,32	6,19	4x	12x
	De_10_2	4x	2,58	3,95	3,87	4x	6x

**Devečanske stijene**	De_10_3	4x	2,58	5,38	5,27	4x	8x
	De_12_1	4x	2,58	5,39	5,28	4x	8x
	De_13_1	4x	2,55	6,79	6,65	6x	10x
	De_13_2	4x	2,54	3,90	3,82	4x	6x
	De_13_3	4x	2,58	6,68	6,55	4x	10x
	De_13_4	4x	2,65	6,51	6,38	4x	10x
	De_14_1	4x	2,56	6,45	6,32	4x	10x
	De_14_10	4x	3,93	6,88	6,74	6x	10x
	De_14_2	4x	3,93	6,91	6,78	6x	10x
	De_14_3	4x	2,62	6,68	6,55	4x	10x
	De_14_4	4x	2,63	4,01	3,93	4x	6x
	De_14_5	4x	2,62	4,28	4,19	4x	6x
	De_14_6	4x	2,62	6,60	6,47	4x	10x
	De_14_9	4x	2,62	4,06	3,98	4x	6x
	De_15_1	4x	2,54	6,51	6,38	4x	10x
	De_15_10	4x	2,54	7,74	7,59	4x	12x
	De_15_11	4x	2,58	7,74	7,59	4x	12x
	De_15_12	4x	2,51	7,73	7,58	4x	12x
	De_15_13	4x	2,55	3,90	3,82	4x	6x
	De_15_14	4x	2,55	7,74	7,58	4x	12x
	De_15_15	4x	2,58	6,49	6,36	4x	10x
	De_15_2	4x	3,77	6,42	6,29	6x	10x
	De_15_3	4x	2,54	3,95	3,87	4x	6x
	De_15_4	4x	2,55	6,49	6,36	4x	10x
	De_15_5	4x	2,58	6,58	6,44	4x	10x
	De_15_6	4x	2,44	3,95	3,87	4x	6x
	De_15_7	4x	2,58	3,89	3,81	4x	6x
	De_15_8	4x	2,58	3,95	3,87	4x	6x
	De_15_9	4x	2,58	7,95	7,79	4x	12x
	De_19_1	4x	2,48	5,68	5,57	4x	9x
	De_19_2	4x	2,55	6,60	6,47	4x	10x
	De_19_3	4x	2,58	6,41	6,28	4x	10x
	De_19_4	4x	2,65	7,95	7,79	4x	12x
	De_19_5	4x	2,60	7,48	7,33	4x	10x
	De_19_6	4x	2,55	7,64	7,49	4x	12x
	De_19_7	4x	2,52	6,42	6,29	4x	10x
	De_1A_1	4x	2,61	8,06	7,90	4x	12x
	De_1A_2	4x	2,57	4,00	3,92	4x	6x
	De_2_1	4x	2,55	3,83	3,76	4x	6x
	De_2_2	4x	2,62	7,05	6,91	4x	11x
	De_2_3	4x	2,54	7,44	7,29	4x	12x
	De_2_4	4x	2,55	7,26	7,11	4x	11x
	De_2_5	4x	2,62	5,39	5,28	4x	8x
	De_3A_1	4x	2,65	6,76	6,62	4x	10x
	De_4A_1	4x	2,65	7,95	7,79	4x	12x
	De_4A_2	4x	2,58	3,95	3,87	4x	6x
	De_4B_1	2x	1,42	1,93	1,89	2x	3x
	De_4B_3	2x	1,34	2,01	1,97	2x	3x
	De_5A_1	4x	2,65	4,05	3,97	4x	6x
	De_5A_2	4x	2,55	7,84	7,69	4x	12x
	De_5A_3	4x	2,57	6,59	6,45	4x	10x
	De_6A_1	4x	2,64	6,67	6,54	4x	10x
	De_7A_1	4x	2,58	6,76	6,63	4x	10x
	De_7A_2	4x	2,64	4,00	3,92	4x	6x
	De_7A_3	4x	2,55	3,89	3,82	4x	6x
	De_7A_4	4x	2,61	7,96	7,80	4x	12x
	De_7A_5	4x	2,51	6,50	6,37	4x	10x
	Vo_1_1	4x	2,58	6,49	6,36	4x	10x
**Vošac**	Vo_1_2	4x	2,58	6,30	6,18	4x	10x
	Vo_1_3	4x	2,41	3,84	3,77	4x	6x
	Vo_1_4	4x	2,58	6,23	6,11	4x	9x

Vo_1_6	4x	2,50	6,15	6,03	4x	10x
Vo_11_1	4x	2,54	7,74	7,58	4x	12x
Vo_11_10	4x	2,46	3,80	3,73	4x	6x
Vo_11_11	4x	2,51	7,74	7,59	4x	12x
Vo_11_12	4x	2,51	3,90	3,82	4x	6x
Vo_11_13	4x	2,51	3,84	3,76	4x	6x
Vo_11_14	4x	2,48	6,26	6,13	4x	10x
Vo_11_15	4x	2,49	7,57	7,42	4x	12x
Vo_11_16	4x	2,50	6,46	6,34	4x	10x
Vo_11_17	4x	2,46	7,58	7,43	4x	12x
Vo_11_18	4x	2,51	7,16	7,02	4x	11x
Vo_11_19	4x	2,48	6,33	6,20	4x	10x
Vo_11_20	4x	2,46	6,36	6,23	4x	10x
Vo_11_21	4x	2,48	7,57	7,42	4x	12x
Vo_11_22	4x	2,51	7,74	7,59	4x	12x
Vo_11_23	4x	2,48	7,57	7,42	4x	12x
Vo_11_24	4x	2,51	7,74	7,59	4x	12x
Vo_11_25	4x	2,61	7,84	7,68	4x	12x
Vo_11_26	4x	2,48	7,65	7,49	4x	12x
Vo_11_27	4x	2,54	7,74	7,58	4x	12x
Vo_11_28	4x	2,52	6,42	6,29	4x	10x
Vo_11_29	4x	2,54	7,74	7,58	4x	12x
Vo_11_3	4x	2,58	6,32	6,19	4x	10x
Vo_11_30	4x	2,51	7,14	7,00	4x	11x
Vo_11_31	4x	2,54	7,44	7,29	4x	12x
Vo_11_4	4x	2,58	7,54	7,39	4x	12x
Vo_11_5	4x	2,51	6,41	6,28	4x	10x
Vo_11_6	4x	2,51	6,08	5,96	4x	10x
Vo_11_7	4x	2,55	6,50	6,37	4x	10x
Vo_11_8	4x	2,54	7,85	7,69	4x	12x
Vo_11_9	4x	2,54	6,51	6,38	4x	10x
Vo_12_1	4x	2,61	7,84	7,68	4x	12x
Vo_12_10	4x	2,58	7,54	7,39	4x	12x
Vo_12_11	4x	2,55	7,84	7,68	4x	12x
Vo_12_12	4x	2,44	7,53	7,38	4x	12x
Vo_12_13	4x	2,51	6,32	6,19	4x	10x
Vo_12_14	4x	2,44	3,84	3,76	4x	6x
Vo_12_15	4x	2,55	3,83	3,76	4x	6x
Vo_12_16	4x	2,54	6,41	6,28	4x	10x
Vo_12_17	4x	2,61	4,00	3,92	4x	6x
Vo_12_18	4x	2,61	8,07	7,91	4x	12x
Vo_12_19	4x	2,61	7,63	7,48	4x	12x
Vo_12_2	4x	2,57	7,84	7,68	4x	12x
Vo_12_20	4x	2,58	6,32	6,20	4x	10x
Vo_12_3	4x	2,55	4,00	3,92	4x	9x
Vo_12_4	4x	2,58	6,41	6,28	4x	10x
Vo_12_5	4x	2,54	7,85	7,69	4x	12x
Vo_12_6	4x	2,57	6,59	6,45	4x	10x
Vo_12_7	4x	2,54	7,74	7,58	4x	12x
Vo_12_9	4x	2,54	7,54	7,39	4x	12x
Vo_2_3	4x	2,54	6,16	6,03	4x	10x
Vo_3	4x	2,62	8,01	7,85	4x	12x
Vo_7_1	4x	2,57	6,15	6,02	4x	9x
Vo_7_2	4x	2,55	4,28	4,19	4x	7x
