# Supplementary material for: Genome Size, Cytotype Diversity and Reproductive Mode Variation of Cotoneaster integerrimus (Rosaceae) from the Balkans
Source: Plants (Basel). 2021 Dec 17;10(12):2798. doi: 10.3390/plants10122798 (PMC8708406; doi:10.3390/plants10122798)
Supplement: Supplementary file 1 [file plants-10-02798-s001.zip › Table S2. Genome composition at five microsatellite loci or Cotoneaster integerrimus_Bogunic et al_Plants.pdf]

Table S2. Genome composition of the studied *Cotoneaster integerrimus* individuals at five microsatellite loci. The numbers in columns denote allele sizes at each locus; MLG - multilocus genotype.

**Site ID:** individual ID numbers	**Number of individuals sharing the MLG**	**Ploidy level**	**Microsatellite loci**																			
			**MSS5**				**CH01F02**				**CH02D11**				**MSS16**				**CH01H10**			
**Go:** 1, 6, 8, 11, 12, 13, 14, 16, 19	9	4x	134	140	142	146	198	212	214	216	159	161			152	154	158	176	106	108	116	
**Go:** 15	1	4x	134	140	142	146	198	212	214		169	171			152	154	158	176	106	110	116	
**Bg:** 1, 2, 3, 4, 5, 6, 8, 9, 10, 11, 12, 13, 14, 15, 16, 17, 18, 19, 20, 21, 3; **Vo:** 11; **Go:** 3, 4, 5, 7, 9, 10, 17, 18, 20; **Ru:** 6, 7, 9, 11, 13, 15, 18, 19, 20; **So:** 4, 7.; **Um:** 8	43	4x	118	132	142	146	204	208	212		169	171			152	154	158	176	108	114	124	
**Um:** 1	1	2x	134	156			214				167				156	158			116	128		
**Um**: 2	1	4x	134	140	142	148	198	204	212	216	161	167			160	162	166	180	106	108	110	
**Um:** 6, 17	2	4x	134	140	142	148	198	204	212	216	161	167			160	162			108	114		
**Um:** 3	1	2x	140	152			214	218			173				154	184			104			
**Um:** 4	1	3x	134	138	146		206	216			159	161	177		160	162			104	110	114	
**Um:** 5	1	3x	134	150	156		198	214			161				154	176			100	110		
**Um:** 7	1	3x	132	142	156		204				167	177			154	166	178		104	112	120	
**Um:** 10	1	2x	130	146			212				169				158	160			106			
**Um**: 11	1	2x	124	148			210				169				154	156			110			
**Um:** 12	1	4x	132	140			198	204	212	220	161	167			160	162	166	180	108	112		
**Um:** 14	1	4x	134	140	142	148	198	204	212	216	161	167			158	160	166	178	110	112		
**Um:** 15	1	2x	132	136			204	214			167	189			154				118			
**Um:** 19; **So:** 1, 2, 3, 16; **Vr**: 6, 12, 13	8	4x	142	146	150		204	206	212		169				154	156	160	176	108	112	114	122
**Um:** 21	1	3x	136	138	142		212	214	216		161	183			154	158	178		110	112	114	
**Um**:1B	1	2x	116	136			210	220			167	171			154				100	108		
**Um:** 2B	1	2x	146				204	218			163	179			156				110	112		
**Um:** 3B	1	2x	156	164			214				167	185			160	184			126	128		
**Um:** 4B	1	2x	146				214	220			169	171			154				104	128		
**Um:** 5B	1	4x	134	138	142	148	198	204	212	216	161	167			158	160	166	178	106	110		
**Um:** 7B	1	2x	132	136			202	204			163	173			154				102	118		
**Um:** 8B	1	2x	118	136			210	220			167	171			154				100	108		
**Vr:** 1, 2, 3, 4, 7, 8	6	4x	134	140	142	148	198	204	212	216	161	167			158	160	166	176	108	112		
**Vr:** 5	1	4x	118	132	142	146	204	208	212		161	167			158	160	166	176	108	114		
**Vr:** 9	1	4x	140	146			204	208	214	216	161	167			154	156	166	176	108	112	116	
**Vr:** 10	1	4x	134	144	146		212	214	216	226	161	167			154	156	160	166	106	116		
**Vr:** 14	1	4x	134	140	142	148	198	204	212	216	161	167			160	162	166	176	106	110		
**Vr:** 15, 16, 17, 18, 19	5	4x	134	140	142	148	198	204	212	216	161	167			160	162	166	176	108	112		

**Bo**: 1, 4, 6, 7, 8, 15, 16	7	4x	142	146	148	152	204	206	212		169	173		152	154	158	176	108	110	114	122
**Bo**: 3, 9, 14	3	4x	142	146	148	150	204	206	212		169	173		152	154	158	176	110	112	114	120
**Bo**: 5, 10, 18	3	4x	142	146	148	152	204	206	212		169	173		152	154	158	176	108	110	114	118
**Bo**: 11, 17	2	4x	140	146	148	152	204	206	212		169	173		152	154	158	176	108	110	114	120
**Bo**: 19	1	4x	142	144	146		204	206	212		169	173		152	154	158	176	108	110	114	118
**Ru**: 1, 2, 3, 4, 5, 14, 16	7	4x	142	146	148		204	208	212		169	171		152	154	158	176	108	112	114	120
**Ru**: 8	1	4x	118	132	142	146	204	208	212		169	171		152	176	182		108	112	114	120
**Ru**: 17	1	4x	142	146	148		204	208	212		169	171		158	160	176		108	112	122	
**Bg**: 7	1	4x	124	132	142	146	204	208	212		169	171		154	156	160	176	108	114		
**So:** 5, 9, 10, 11, 13	5	4x	118	132	142	146	204	208	212		169	171		152	154	158	176	108	114		
**So:** 6	1	4x	142	146	150		204	206	212		169	173		154	156	160	176	108	110		
**So:** 7	1	4x	130	146			204	212	216		169	173		154	156	160	176	108	110		
**So:** 14	1	3x	136	146	150		204	206	212		169	171		154	156	176		106	112		
**Pu:** 1, 3, 4, 5, 6, 7, 8, 9, 10, 11, 12, 13, 14, 15, 16, 17, 18, 19, 20; **Ca**: 20	20	4x	134	140	142	146	198	204	212	214	159	161		154	156	160	176	104	108		
**Ca**: 2	1	4x	134	140	148		198	204	212	216	159	167		156	160	166	176	106	110		
**Ca:** 3, 4, 5, 6, 7, 8, 9, 10, 11, 12, 13, 14, 16, 17, 18, 19, 3	17	4x	134	140	148		198	204	212	216	159	167		158	162	166	176	106	110		
**Ca**: 15	1	4x	118	130	140	148	198	204	212	216	161	167		158	162	166	176	106	110		
**Ca**: 21	1	4x	134	140	142	146	198	204	212	214	159	161		154	156	160	176	104	108	114	
**De**: 1, 2	2	4x	118	130	134	142	200	204	206	216	161	167		154	160			106	110	116	
**De:** 3, 4, 6, 8, 9, 10, 11, 12, 14, 15, 20	11	4x	132	142	148	156	204	216			165	177		154	158	166	178	104	112	118	
**De:** 5	1	4x	118	130	140	142	204				161	167		154	160			106	110	116	
**De:** 7, 13, 21	3	4x	134	140	146	150	198	206	216		161	177		162				106	116		
**De**: 16	1	2x	138	146			204	216			161	173		156				104	118		
**De**: 19	1	4x	132	142	148	156	204	216			165	177		154	158	166	178	104	120		
**Rt:** 1, 2, 3, 10	4	4x	130	132	148	152	204	214	220		165	175		156	158	176		104	112	124	
**Rt:** 4, 5, 6	3	4x	130	132	154		198	214	220	226	153	159		154	166	178		108	112	118	120
**Rt:** 7	1	4x	144	150			204	208	214	222	161	163		156				104	108		
**Rt:** 9	1	3x	116	132	142		204	208	212		161	163		154				104	108		
**Mu**: 1, 3, 5, 6, 7	5	4x	124	140	152		194	204	212		153	175		154	180			106	112	120	
**Mu:** 2, 4	2	4x	126	132	140	150	198	204	214	220	151	175		154	156	164	176	108	110	112	
**Vo:** 1, 3, 7, 8, 9, 10	6	4x	144	146	148		204	206	212		169	173		154	156	160	176	106	110	114	118
**Vo:** 5	1	4x	150	152	154	156	204	208	212		169	173		154	158	176		106			
**Vo:** 6	1	3x	116	132	142		204	208	212		169	173		154	158	176		106			
**Vo:** 11	1	4x	142	146			204	206	212		169	173		154	156	160	176	104	106	112	118
**Si:** 1, 2,3, 4, 5	5	4x	140	144	146	150	204	216	218		137	161		158	162	166		102			
**Pr:** 1	1	3x	134	138	140		198	204	214		159	161		154	160	178		108			
**Pr:** 2, 4	2	4x	132	140	146	156	212	214	216	222	161	163	167	156	160	162		98			
**Pr: 3**	1	3x	134	138	142		198	204	216		159	161		154	160	178		106			

**He:** 1, 2, 3, 4, 5	5	4x	126	128	134		198	204	212	218	155	159			154	164	172	174	84			
**Ro**: 1	1	4x	130	140	142	146	198	202	212	218	163	165	167		156	160	162	166	104	106	110	
**Ro**: 2, 4, 5	3	4x	130	140	144		198	204	212	218	165	169			156	160	162	166	104	106	110	
**Ro**: 3	1	4x	132	140	146	156	202	212	214	222	161	163	167		156	160	162		98	104	106	
**Mo:** 1, 3	2	4x	128	132	136		194	198	204	214	153	175			154	166	178	194	104	108	112	122
**Mo:** 2	1	4x	134	138	144	152	198	204	212	216	163	169			156	160	162	176	102	104	106	114
**Mo:** 4	1	4x	134	138	144	152	198	204	212	216	163	169	171		160	162	176	192	102	106	116	
**Su:** 1	1	4x	118	132	140	142	202	206	210	230	139	161	185		156	160	176	188	112	114	126	
**Su:** 2	1	4x	118	132	140	142	202	206	210	230	139	151	163	181	158	164	180		108	112	124	
**Su:** 3	2	4x	118	134	140	146	202	206	210	220	139	161	185		156	160	176	188	112	114	122	
**Su:** 4, 5	1	4x	118	132	140	142	202	206	210	230	139	161	185	199	156	160	176	188	112	114	126	
**Su:** 6, 7	1	4x	118	132	142	146	202	206	210	230	139	163			156	180			106	112	124	
**Ol:** 1	1	4x	118	132	140	142	202	206	210	230	159	191	193	197	156	194			104	112		
**Ol:** 2	1	4x	118	132	142	144	202	206	210	230	197	199			156				110	114		
**Ol:** 3	1	4x	118	132	140	142	202	206	210	230	139	151	181		156				114			
**Ol:** 4, 5	2	4x	118	132	142	144	202	206	210	230	139	159			154	156			122			
**Pa:** 1	1	4x	118	132	142	146	202	206	210	230	131	175	191		156	160	180		110	122		
**Pa:** 2	1	4x	118	132	142	146	202	206	210	230	139	175	191		156	160	180		106	114	122	
**Pa:** 3	1	4x	118	132	142	146	202	206	210	230	177				160	194			110	112	116	
**Pa:** 4, 5	2	4x	118	132	144	152	202	206	210	230	157	163	217		154	156	170	190	110	112	120	
