# Supplementary figures and images for: Genome Size, Cytotype Diversity and Reproductive Mode Variation of Cotoneaster integerrimus (Rosaceae) from the Balkans
Source: Plants (Basel). 2021 Dec 17;10(12):2798. doi: 10.3390/plants10122798 (PMC8708406; doi:10.3390/plants10122798)

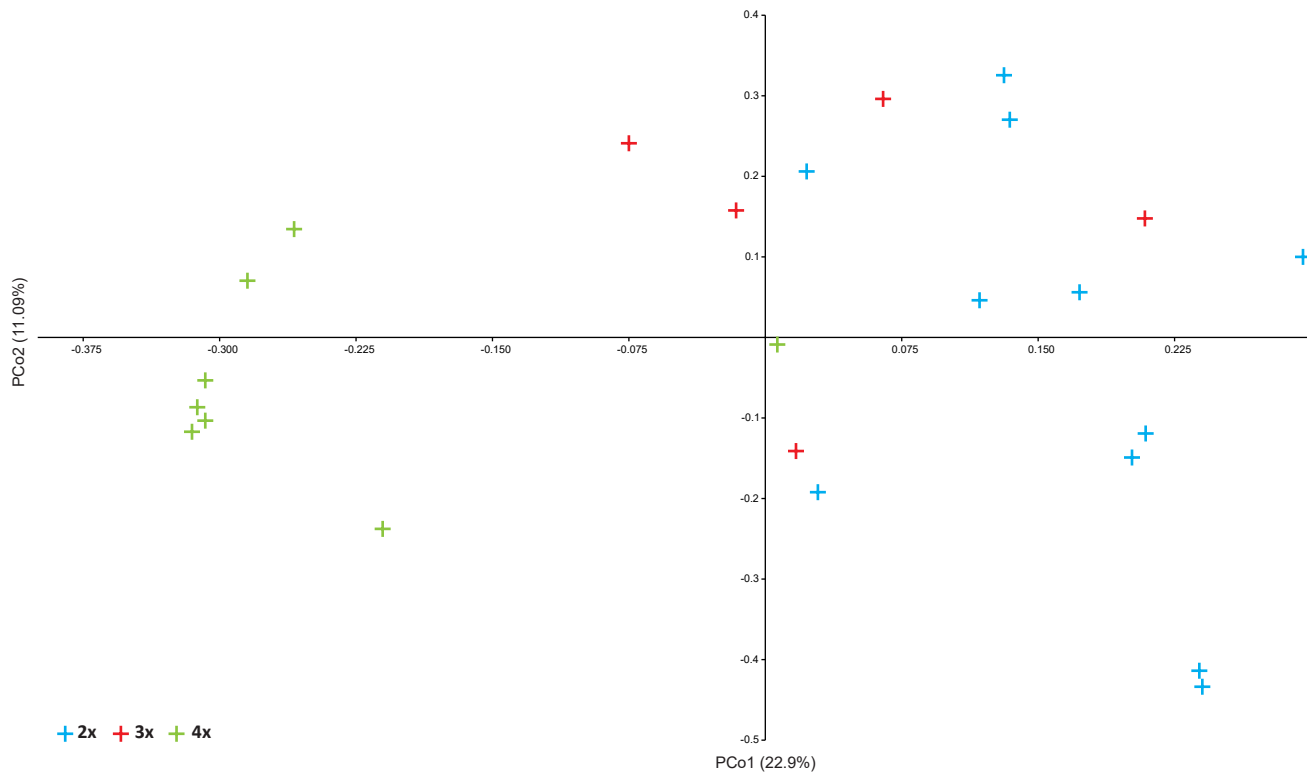

Supplement: Supplementary file 1 [file plants-10-02798-s001.zip › Figure S1. PCoA_Cotoneaster integerrimus MLGs_Bogunic et al_2021_Plants.pdf]
